# *Macrotene* chromosomes provide insights to a new mechanism of high-order gene amplification in eukaryotes

**DOI:** 10.1038/ncomms7154

**Published:** 2015-01-30

**Authors:** Agnès Thierry, Varun Khanna, Sophie Créno, Ingrid Lafontaine, Laurence Ma, Christiane Bouchier, Bernard Dujon

**Affiliations:** 1 Institut Pasteur, Unité de Génétique moléculaire des levures, CNRS UMR3525, Sorbonne Universités, UPMC, Univ. Paris 06 UFR927, 25, rue du Docteur Roux, F-75724 Paris, France; 2 Institut Pasteur, Genomic platform, 28, rue du Docteur Roux, F-75724 Paris, France

## Abstract

Copy number variation of chromosomal segments is now recognized as a major source of genetic polymorphism within natural populations of eukaryotes, as well as a possible cause of genetic diseases in humans, including cancer, but its molecular bases remain incompletely understood. In the baker’s yeast *Saccharomyces cerevisiae*, a variety of low-order amplifications (segmental duplications) were observed after adaptation to limiting environmental conditions or recovery from gene dosage imbalance, and interpreted in terms of replication-based mechanisms associated or not with homologous recombination. Here we show the emergence of novel high-order amplification structures, with corresponding overexpression of embedded genes, during evolution under favourable growth conditions of severely unfit yeast cells bearing genetically disabled genomes. Such events form massively extended chromosomes, which we propose to call *macrotene,* whose characteristics suggest the products of intrachromosomal rolling-circle type of replication structures, probably initiated by increased accidental template switches under important cellular stress conditions.

## Abstract (web summary)

Copy number variation is an important source of genetic variation in natural populations and may have a role in human disease. Here, the authors identify high-order amplification structures that form large extended chromosomes and suggest that these may occur due to accidental template switching in stress conditions.

## Graphical abstract

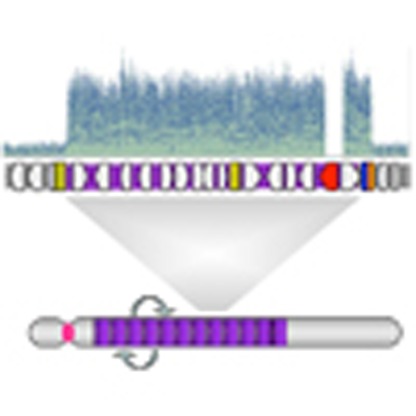

Understanding the molecular mechanisms at the basis of genome maintenance and dynamics is not only a fascinating question of biological evolution but it also bears great potential interest for the prevention or the cure of major genetic diseases in humans. Remarkable progress along these lines were recently made possible by the application of whole-genome sequencing techniques to analyse evolutionary experiments on unicellular organisms^1^ or to characterize human cancer cells^2^,^3^. Next to *Escherichia coli*, on which experimental evolution was pioneered in *Bacteria*^4^, the baker’s yeast *Saccharomyces cerevisiae* offers equivalent advantages for eukaryotes. The rate of nucleotidic mutation was determined over its entire genome under normal growth conditions^5^ and the mutational landscape of some mutator strains has been characterized^6^. Evolutionary experiments aimed at studying adaptation to controlled selective pressures^7^,^8^,^9^,^10^,^11^,^12^ or the recovery from artificial gene dosage imbalance^13^,^14^,^15^ revealed the frequent formation in this genome of low-order segmental amplifications (mostly two times, rarely up to four or five times), in addition to the more classical point mutations in genes or their regulatory elements. These amplifications exhibit a variety of topological forms such as intra- or inter-chromosomal segmental duplications, ranging in size from a few^9^ to hundreds of kilobases^13^,^14^, or supernumerary chromosomes (neochromosomes), made of two large segmental duplications joined together^13^. Episomes were also reported, propagating short chromosomal segments in independent circular^10^,^15^,^16^ or linear forms^11^,^17^.

These experimentally generated structures show variable levels of instability^18^ and tend to disappear over successive generations when the original selective factor is eliminated. Yet, segmental duplications are prone to play a prominent role in modelling genomes over large evolutionary timescales^19^, and traces of intra- and interchromosomal segmental duplications are observed in natural genomes of a variety of eukaryotic organisms, including human where they may occasionally have pathogenic consequences^20^. In natural yeast isolates, such traces are generally scarce, except in subtelomeric regions^21^,^22^. But, in the original collection of yeast deletion mutants, large segmental duplications and whole-chromosome aneuploidy were frequently observed with, for some of them, demonstrated phenotypic effects^23^. Such structures may also represent transient evolutionary solutions for long-term adaptation to stressful environmental conditions^24^. Several mechanisms have been invoked at the origin of these temporary low-order amplification structures, including non-allelic homologous recombination between dispersed repeated sequences made of transposable elements or their remnants^10^,^15^,^25^, micro-homology-mediated repair of accidentally broken replication forks^14^ and interference between adjacent replication origins^26^. Replication stress and loss of replication control are potent inducers of these mechanisms^27^,^28^,^29^.

By contrast to the above, high-order gene amplifications (over ca ten times) are much more rare in eukaryotic genomes despite the classical example of the methotrexate resistance in *in-vitro* cultivated mammalian cells^30^, and a few normal gene clusters such as those encoding rRNAs or histones^31^. In *S. cerevisiae*, the tandem gene array at the *CUP1* locus can expand to dozens of copies after selection for resistance to toxic copper salts^32^,^33^, but the repeat unit being of small size (2 kb), this amplification has only limited effects on the overall chromosome size. The same is true for the tandem repeats of the hexose transport loci *HXT6* and *HXT7* selected in response to glucose-limited environments^34^.

In most evolutionary experiments performed so far, the focus was placed on studying the response of normal yeast genomes to limiting or deleterious environmental conditions, rather than examining spontaneous mutational events in genomes in the absence of external selection. In particular, very little is known so far on the evolutionary trajectories followed by severely altered eukaryotic genomes, resulting from accidental mutations, when cells are allowed to proliferate under optimal conditions, as is the case in some cancers. To examine this question, we built permanently disabled *S. cerevisiae* genomes by replacing key essential genes by their orthologues from another yeast species, taking advantage of the large evolutionary spectrum offered by presently sequenced *Saccharomycotina* genomes^21^. Viable haploid and diploid *S. cerevisiae* strains were obtained in which either the asparaginyl-tRNA synthetase gene (*DED81*) or the lysinyl-tRNA synthetase gene (*KRS1*) was precisely replaced by its orthologue from *Yarrowia lipolytica*, a very distantly related yeast species^35^,^36^. These transgenic strains grew extremely slowly even in rich glucose medium but retain their ability to propagate indefinitely by mitosis, as well as to mate and sporulate. During prolonged cultures of these strains in rich medium, a variety of faster growing mutants spontaneously appeared, among which we observed novel high-order amplification structures extending chromosome sizes by over 1.5 times (*macrotene* chromosomes).

We report here the detailed molecular analysis of these unexpected structures, whose unusual characteristics compared with previously described gene amplifications in eukaryotes suggest that they resulted from uncontrolled replication of long chromosomal segments after accidental template switching events, a phenomenon possibly triggered by the severe functional stress imposed on our genetically engineered cells.

## Results

### Evolutionary experiments

**Figure 1:**
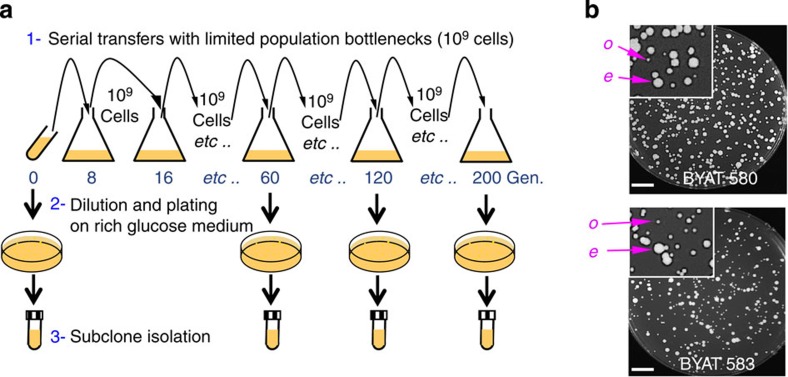
Scheme of evolutionary experiments. (**a**) Severely unfit diploid strains BYAT580 and BYAT583 were grown in YPD medium at 30 °C in 2-l cultures with rotational shaking. At the end of each culture (see Tables 1 and 2 for numerical details), an aliquot of 10^9^ cells was inoculated in 2 l of fresh medium. Another aliquot was plated on solid YPD medium (after appropriate dilution) and incubated at 30 °C for 3–6 days to obtain subclones. The process was serially repeated 23 times. (**b**) Examples of growth heterogeneity of subclones during evolution of populations (*o:* original colony size, *e:* evolved mutant). Scale bar, 1 cm.

The construction and phenotypic characterization of the transgenic *S. cerevisiae* strains bearing the *Y. lipolytica* orthologues of, respectively, *DED81* (chromosome VIII) or *KRS1* (chromosome IV) are detailed in Supplementary Notes 1,2 and 3 and Supplementary Figs 1 and 2. Diploid strains BYAT580 and BYAT583 (*DED81* and *KRS1* gene replacements, respectively, Supplementary Table 1) were cultivated at 30 °C in rich glucose medium (YPD) for a total of ca 200 successive generations by a serial-transfer protocol, keeping the population bottlenecks at 10^9^ cells (Fig. 1 and Tables 1, 2). The first cultures grew extremely slowly, as expected from a poor adaptation of the *Y. lipolytica* tRNA synthetases to the pools of cognate *S. cerevisiae* tRNA molecules (Supplementary Fig. 3). But, as cultures evolved, faster growing mutants appeared that eventually overcame the populations despite long-term persistence of colony size heterogeneity. Colonies (subclones) of initial size or larger (evolved mutants) were picked up at various intervals (Tables 1 and 2) and analysed by a variety of molecular techniques including, for some of them, whole-genome sequencing (Methods).

### Nested amplicons identified from whole-genome sequencing

**Figure 2:**
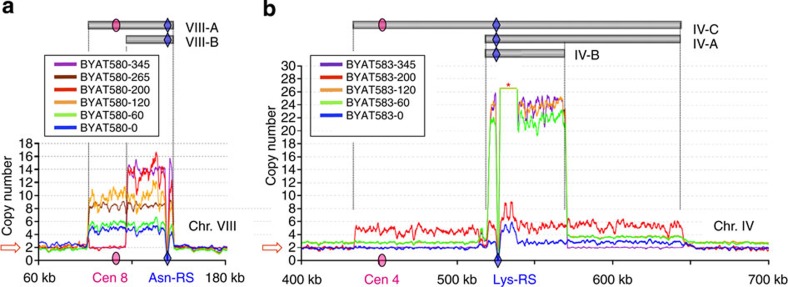
Amplicons identified from genome sequencing coverages. (**a**) Copy number variation along a chromosome VIII segment, as determined from sequence coverages of parental and evolved strains of the BYAT580 evolutionary experiment (see Methods). For strain numbering, refer to Table 1. BYAT580-265 and -345 are subclones of BYAT580-120 and -200, respectively, after 145 additional generations (see the text and Supplementary Fig. 10). Copy numbers (ordinates) were normalized to 2 (for diploids). Horizontal grey bars on top materialize amplicon units. Abrupt curve drops correspond to the *Y. lipolytica* Asn-RS gene (blue diamond) absent from the *S. cerevisiae* reference sequence. Pink oval: centromere. (**b**) Copy number variation along a chromosome IV segment, as determined from sequence coverages of parental and evolved strains of the BYAT583 evolutionary experiment. Same legend as in **a**, BYAT583-345 is a mutant subclone of BYAT583-201 (Table 1) after 145 additional generations (see the text and Supplementary Fig. 10). Blue diamond: *Y. lipolytica* Lys-RS gene. *Location of *ENA* tandem gene array (artificially cut for clarity). Note the complex pattern of amplifications along chromosome IV because of the superposition of several events.

In the BYAT580 evolutionary experiment, two nested amplicon units were identified from genome sequencing (Fig. 2a). The 55-kb-long amplicon VIII-A appeared in four and eight copies in excess to diploid number in the first two evolved mutants sequenced, BYAT580-60 and -120, respectively. Note its presence in three extra copies in BYAT580-0, indicating a very early mutational event, and its conservation (in 6 extra copies) in subclone BYAT580-265 (see below), indicating long-term stability. The 30-kb-long amplicon VIII-B accumulated in 12 extra copies in the late-appearing mutant BYAT580-200, and was also conserved in its subclone, BYAT580-345, isolated after additional generations (see below). For precise limits of both amplicons, see Supplementary Fig. 4a. Amplicon VIII-A contains the centromere, the *YALI* Asn-RS gene and all *S. cerevisiae* genes (29 Coding Sequences -CDS- and 4 tRNA genes - tDNA) from coordinates 91,387 to 146,472 along with chromosome VIII. Amplicon VIII-B shares external limits with amplicon VIII-A but lacks the internal centromeric region containing 14 CDSs and 1 tDNA, see below). No other copy number alteration or aneuploidy was observed in the rest of the genomes of these strains (Supplementary Fig. 5a).

In the BYAT583 evolutionary experiment, three nested amplicon units were identified on chromosome IV, whose simultaneous presence in some strains generated more complex patterns (Fig. 2b). A high-level amplification (20–22 copies in excess to diploid number) of a 50-kb-long centromere-less segment (amplicon IV-B) appeared in early-arose mutants (BYAT583-60 and -120). It is also visible in similar numbers in the independently arose mutant BYAT583-345 (see below). Amplicon IV-B contains the *YALI* Lys-RS gene and 23 CDS (including the *ENA* tandem repeat), 3 tDNA and 1 small nuclear RNA gene of *S. cerevisiae* (Supplementary Fig. 4b). By contrast, the 211-kb-long amplicon IV-C, overlapping the centromere, was only observed (in three extra copies) in the late-appearing mutant BYAT583-200. It contains the *YALI* Lys-RS gene, the centromere and all *S. cerevisiae* genes (99 CDS, plus 8 tDNAs and 1 snoRNA gene) from coordinates ca 434,600 to ca 645,600 (Supplementary Fig. 6c). The 132-kb-long centromere-less amplicon IV-A corresponds to a segmental duplication between two Ty elements (Supplementary Fig. 6b), which occurred in one of the two copies of chromosome IV in strain BYAT583-0 and propagated to evolved mutants BYAT583-60, -120 and -200 without providing much novelty compared with already known segmental duplication structures^19^. Note, however, that the two elements (*YDRCTy2-1* and *YDRCTy1-1*) belong to distinct families and share only 78% nucleotide sequence identity. This segmental duplication, absent from BYAT583-345, is an independent event from other amplifications. Finally, the BYAT583 evolutionary trajectory was further complicated by the transient formation of a chromosome IV disomy (in strains BYAT583-60 and -120, Supplementary Fig. 5b), a phenomenon probably related to functional stress^24^ but also independent from the high-order amplifications studied here (also absent from BYAT583-345).

### Determination of amplification structures

**Figure 3:**
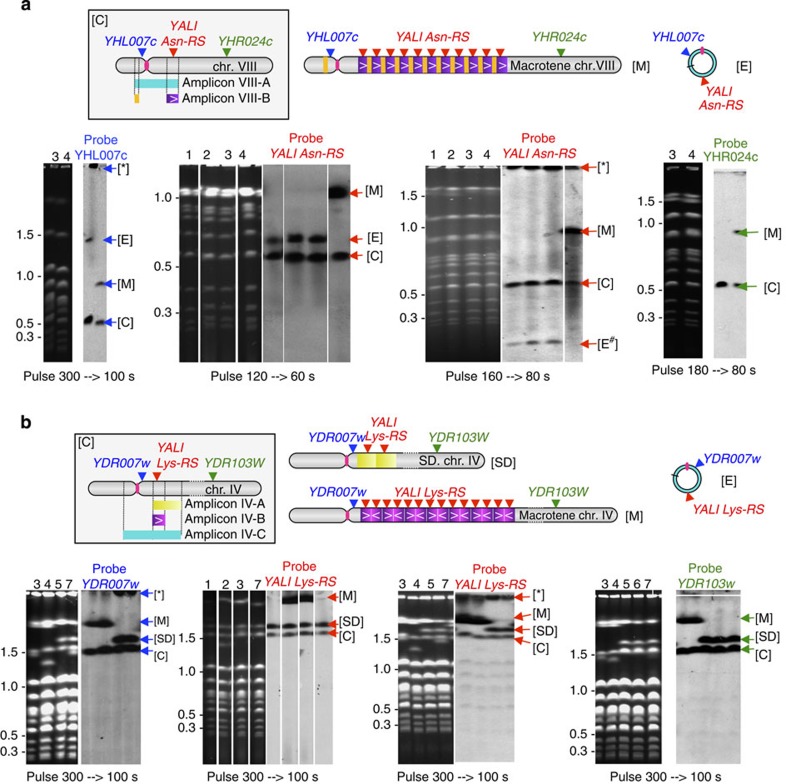
Pulsed-field gel electrophoresis of parental and evolved strains. PFGE were run according to Methods with pulse ramps as indicated (linear accelerations). Left: ethidium bromide fluorescence. Size scale in Mb, calibrated from migration of natural *S. cerevisiae* chromosomes. Right: hybridization with indicated probes. (**a**) BYAT580 evolutionary experiment. Top diagrams: location of probes with respect to amplicons (box) and scheme of amplified structures observed: [C] normal chromosome (scheme in box), [M] *macrotene* chromosome (arbitrary repeat number), [E] circular episome, [*] hybridizing material in slot (including large circular episomes), [E#] broken episome (linearized DNA). Strains 1: BYAT580-0, 2: BYAT580-60, 3: BYAT580-120, 4: BYAT580-200. Note the variable migration of the circular episome relative to chromosomes with different pulse frequencies^63^. (**b**) BYAT583 evolutionary experiment. Same legend as explained in **a**, [SD] segmental duplication. Strains 1: BYAT583-0, 2: BYAT583-60, 3: BYAT583-120, 4: BYAT583-130, 5: BYAT583-170, 6: BYAT583-190, 7: BYAT583-200.

To determine the structure of the amplifications identified above, we ran pulsed-field gel electrophoreses (PFGE) of intact chromosomal DNA from parental and evolved strains (Fig. 3). In each evolutionary experiment, the mutants with highest amplification levels showed massively extended (*macrotene*) chromosomes. In strain BYAT580-200 (Fig. 3a), one copy of the two chromosome VIII homologues (identified from hybridizing probes) migrated at ca 900 kb size, whereas the other copy remained at its normal 583 kb size (strains were diploid). The size increase is consistent with the presence of 12 extra copies of the 30-kb-long amplicon VIII-B within the *macrotene* chromosome. Other mutants of the same evolutionary trajectory did not exhibit this structure. Instead, they revealed hybridizing bands that did not correspond to visible ethidium bromide-stained chromosomes and whose migration, varying with pulse frequency, suggested circular episomes (see below).

Similarly, a *macrotene* chromosome IV of ca 2,500 kb instead of its normal 1,532 kb size was observed in mutants BYAT583-60 and -120 (Fig. 3b). It is also present in mutant BYAT583-130 (not sequenced). Again, this size increase agrees (within precision limits) with the presence of 20–22 extra copies of the 50-kb-long amplicon IV-B within one copy of chromosome IV. The other two copies (these strains were aneuploids, see Supplementary Fig. 5b) remained intact or increased in size by the 132 kb segmental duplication of amplicon IV-A (above). Mutant BYAT583-200 that showed lower orders of amplification did not exhibit a *macrotene* chromosome (like BYA583-170 and -190, not sequenced). Their 211-kb-long amplicon IV-C was not visible on gel electrophoresis, also suggesting circular molecules.

### Structure of *macrotene* chromosome amplifications

To gain insight into the formation of the *macrotene* chromosomes, the precise identification of junction sequences was necessary. This task was rendered difficult by the abundance of yeast transposable elements (Ty) and their remnants (solo long-terminal repeat (LTR)) in the vicinity of the regions of interest and, for the BYAT583 evolutionary trajectory, by the simultaneous presence of several distinct amplications in some evolved strains. The problem was eventually solved by a combination of genomic digest hybridizations (Supplementary Figs 7 and 8), PCR amplifications around breakpoints and re-analysis of unmapped reads from genome sequencing data of evolved mutants (Methods).

**Figure 4:**
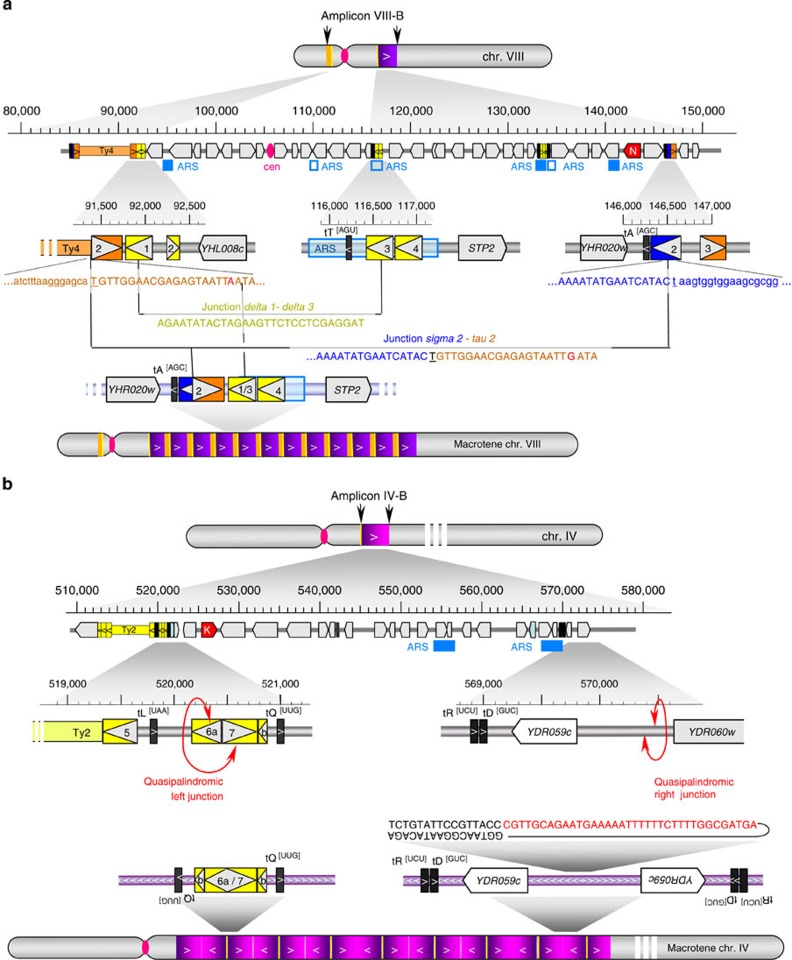
Junction sequences and repeat organizations in *macrotene* chromosomes. (**a**) *Macrotene* chromosome VIII. Grey cylinders with a pink tightning (centromere) schematize normal (top) and *macrotene* (bottom) chromosome VIII. Amplicon VIII-B is symbolized by the yellow (left arm) and purple (right arm) boxes with internal arrows to indicate orientation. The centre part of figure details genetic elements present in the amplicon region (top) with zooms on junction regions (middle). Junction sequences are from Supplementary Fig. 6. Grey arrowhead boxes: protein-coding genes (*Y. lipolytica* Asn*-*RS genes is highlighted in red and labelled with *N*); pink oval: centromere; thick vertical black lines: tRNA genes; yellow, dark blue and orange boxes: *delta*, *sigma* and *tau* elements, respectively (orientation given by internal arrows). Filled or void light blue boxes locate replication origins (ARS), respectively, confirmed or proposed^64^. (**b**) *Macrotene* chromosome IV. Grey cylinders with a pink tightning (centromere) schematize normal (top) and *macrotene* (bottom) chromosome IV. Amplicon IV-B is symbolized by purple boxes of vanishing intensity with internal arrows to indicate orientation. Centre part of figure, same legend as above. The *Y. lipolytica* Lys*-*RS gene is labelled *K*. Sequence of the right junction is indicated (black letters: palindromic part, red letters: unique central part, line indicates direct sequence continuity). Chr., chromosome.

We found that the *macrotene* chromosome VIII was formed by direct tandem repetitions of amplicon VIII-B (Fig. 4a). Remarkably, the junction of the repeat units occurred between two completely different sequences (only one nucleotide (nt) -T- in common) separated from each other by 55 kb (see Supplementary Fig. 9a for details). Between these limits, amplicon VIII-B is internally deleted of a 25-kb segment containing the centromere and adjacent genes. This deletion occurred between two other LTR elements diverged in sequence (*YHLCdelta1* and *YHLCdelta3* sharing 79 % sequence identity) joined together in their longest 29 bp common segment (see Supplementary Fig. 9c for details). *A priori*, the tandem array of amplicon VIII-B could be anywhere in chromosome VIII, including in telomeric regions. Available data, however, favour an *in loco* amplification next to the original segment, as no ectopic junction sequence could be found from restriction mapping (Supplementary Fig. 7) or after re-analysis of whole-genome sequences by a method inspired from the split-read mapping strategy^37^. We are, therefore, left with the idea that amplicon VIII-B was tandemly repeated *in loco* immediately at the right of the original chromosomal copy (the left side is excluded from restriction mapping results).

By contrast, the *macrotene* chromosome IV was formed by inverted tandem repetitions of a continuous, 50-kb-long segment of chromosome IV (amplicon IV-B) with quasi-palindromic junctions (Fig. 4b). This structure was discovered by re-assembly of unmapped sequence reads of BYAT583-345 (Methods). A 77-nt-long contig was obtained whose sequence corresponds to a quasi-palindromic junction formed, in absence of any sequence identity, within the divergent promoter region separating *YDR059c* and *YDR060w*. Existence of this junction was verified by genomic blot hybridizations (Supplementary Fig. 8c). Using the same restriction enzyme-based mapping method, the left boundary of amplicon IV-B was mapped between two inversely oriented LTR elements, *YDRCdelta6a* and *YDRWdelta7,* sharing 77% sequence identity (Supplementary Fig. 8b). This junction could not be resolved at nucleotide level, but mapping is consistent with its occurrence within the longest 42 bp common segment of these two LTRs, forming a second quasi-palindromic junction (a second inverted junction was obviously required to form an array of inverted repeats, but not necessarily a quasi-palindromic one). Again, all available mapping data indicate an *in loco* formation of the repeat array next to the genuine amplicon IV-B segment, on either its right or its left side.

### Structure of other amplifications

To help clarify the origin of *macrotene* chromosomes, we also determined the amplification junctions in the other evolved mutants from the same evolutionary trajectories suspected of containing circular episomes according to PFGE results (Fig. 3), in agreement with the presence of centromeres in amplicons VIII-A and IV-C excluding intrachromosomal amplifications. To demonstrate these structures, we sequenced the novel junctions formed in the mutants, from PCR amplifications on their genomic DNA. In the BYAT583 experiment, we found that amplicon IV-C circularized by junction between two co-oriented solo *delta* elements, *YDLCdelta1* and *YDRCdelta7,* within their 10-nt-long common sequence stretch, forming a 211-kb centromeric episome (Supplementary Fig. 6c). Superficially, this looks similar to previously described short circular episomes in yeast, formed by homologous recombination between dispersed repeats^10^,^15^, except that the two solo *delta* elements involved here are significantly diverged in sequence (79% nucleotide identity) and that this episome is much larger and centromeric. In addition, contrary to previous cases, no corresponding deletion of the cognate chromosomal segment was observed (Fig. 3), arguing against a reciprocal genetic recombination.

In the BYAT580 experiment, we found that amplicon VIII-A formed a 50-kb circular episome (Supplementary Fig. 9b). Ruling out homologous recombination was even simpler in this case because the novel junction occurred between the two completely different sequences *YHLWtau2* and *YHRCsigma2* mentioned above (the same junction as amplicon VIII-B repeats in the *macrotene* chromosome). Again, no corresponding deletion was observed on the chromosome. Formation of this episome must, therefore, have involved another mechanism. Interestingly, there exists a short sequence identical to the episomal junction immediately upstream of the same Ty4 element (because it is inserted next to another inverted *solo* sigma element, *YHRCsigma1*), leading us to the speculative hypothesis that a ‘guide’ or template sequence helped the circularization of amplicon VIII-A (Supplementary Fig. 9a). The minor sequence divergence between the two LTRs of the Ty4 element (a G within *YHLWtau1* at position 85,554 versus an A within *YHLWtau2* at position 91,406, both verified by re-sequencing) supports this hypothesis of ‘guide’ sequence because the G, not the A, was found in the amplified copies of the circular episome (as well as in the *macrotene* chromosome VIII, see Fig. 4). No other *sigma*-*tau* junction in inverted orientation exists in the rest of the *S. cerevisiae* genome.

### Reconstruction of evolutionary trajectories

The presence of mutants bearing circular episomes together with mutants bearing *macrotene* chromosomes in both evolutionary trajectories, albeit in opposite order of appearance, raised the question of the independence of their formation (in our protocol, the evolved mutants sequenced were independent subclones from evolving populations, not derivatives from one another as will be described in next paragraph). We, therefore, analysed additional mutants isolated at different intervals to better describe the mutational trajectories within evolving populations. In the BYAT580 experiment (Table 1), all evolved mutants isolated until generation 130 contained the circular episome (11 in total, in addition to BYAT580-0, -60 and -120). Four other episome-bearing mutants were also isolated from generations 140 to 200, but they cohabited in the evolving population with other mutants bearing either the *macrotene* chromosome alone (1 mutant, in addition to BYAT580-200) or both structures simultaneously (4 mutants). This trajectory leaves open the possibility (and even suggests) that the *macrotene* chromosome VIII arose as a secondary mutation from episome-bearing mutants (see Discussion). By contrast, in the BYAT583 experiment (Tables 1 and 2), all evolved mutants isolated until generation 130 exhibited a *macrotene* chromosome (8 in total, in addition to BYAT583-60 and -120), whereas all mutants studied from generations 150 to 200 contained the episome (5 in total, in addition to BYAT583-200). At generation 140, one mutant of each type was obtained, consistent with an intermediate stage during the evolution of this population whose mutational trajectory excludes the possibility that the *macrotene* chromosome IV arose from pre-existing episomes, a conclusion reinforced by the complete lack of relationship between the molecular structures of amplicons IV-B and IV-C (above).

### Stability of evolved mutants

The stability of mutants bearing *macrotene* chromosomes or episomes was first estimated at population level by cultivation in rich medium for a total of 145 generations, and then independently measured after transformation of subclones by plasmids expressing the *S. cerevisiae* RS genes (Supplementary Fig. 10a).

At population level, both *macrotene* chromosomes IV and VIII proved remarkably stable (Supplementary Fig. 10b). Samples isolated after 35, 70, 110 and 145 generations kept the same profile (a ca 900 kb *macrotene* chromosome plus the normal chromosome) as the original BYAT580-200 strain (see Fig. 3). Similarly, samples isolated after 35 and 145 generations kept the same profile as the original BYAT583-120 strain (a ca 2 Mb *macrotene* chromosome plus the normal chromosome, note by contrast the instability of the additional copy bearing the 132 kb segmental duplication of amplicon IV-A). The stability of both *macrotene* chromosomes could also be verified from the sequencing of subclones BYAT580-345 and BYAT583-265 isolated, respectively, from BYAT580-200 and BYAT583-120 after 145 generations (Supplementary Fig. 10c). The observed stability of *macrotene* chromosomes looks surprising, but is consistent with previously established figures for segmental duplications. Above results on populations and subclones are compatible with a conservation of original structures ranging from 100 % to roughly half this figure. Now, 50% conservation after 145 generations corresponds to a loss of ca 0.5% per cell per generation, that is, a maximal instability more than two orders of magnitude higher than the instability observed for single tandem duplications of similar sizes^18^.

Completely different patterns were observed for the two circular episomes. At population level, the 50-kb episome of BYAT580-120 proved stable as judged from the presence of a weakly hybridizing band (Supplementary Fig. 10b), in addition to the normal chromosome (migration varies with pulse frequency, compare with Fig. 3). This episome stability could also be verified from sequencing of subclone BYAT580-265 isolated from BYAT580-120 after 145 generations (only the copy number varied, see Fig. 2). On the contrary, mutant BYAT583-200 proved extremely instable. Its 211-kb circular episome was not visualized by PFGE, hence its maintenance during the cultivation is unknown, but, astonishingly, a new *macrotene* chromosome IV appeared in the population after 35 generations and remained present after 145 generations (Supplementary Fig. 10b). Note the presence of the 132-kb segmental duplication in one copy of chromosome IV (as in BYAT583-200), indicating that the new *macrotene* chromosome IV appeared concomitantly to a chromosome IV aneuploidy (as was the case in BYAT583-60 and -120). The same phenomenon must have occurred in cultures of mutants BYAT583-201 and -202 (two other episome-bearing subclones isolated at the same time as BYAT583-200, Table 2) as judged from the presence of a *macrotene* chromosome IV in their subclones (respectively, BYAT583-345 and -346) after 145 generations. Given generation times of mutants (below), a possible interpretation for the rapid reformation of *macrotene* chromosome IV would be the frequent loss of the episome (producing severely unfit cells equivalent to parental strains) out of which novel *macrotene* chromosome mutants are strongly selected.

To estimate the role of the phenotypic selection in the maintenance of amplified structures, we transformed evolved mutants with plasmids of the MoBY-ORF collection^38^ containing the Asn-RS or Lys-RS genes of *S. cerevisiae*, as appropriate, and cultivated the transformants for ca 150 generations before subcloning (Supplementary Fig. 10a). The activity of the plasmids used was directly verified by their ability to restore the phenotypes of non-amplified unfit strains in parallel transformations (not shown). For chromosome VIII, both amplified structures (episome and *macrotene* chromosome) remained stable as before (two subclones tested for each, Supplementary Fig. 10c). For chromosome IV, only one of the three subclones tested from the transformant of BYAT583-265 had kept the *macrotene* chromosome, indicating partial stability compatible with above calculations. The stability of the 211-kb episome could not be assessed by PFGE (above), but in the presence of the MoBY-ORF plasmid, BYAY583-200 did not generate novel *macrotene* chromosome mutants (three subclones tested), in agreement with above interpretation.

### Growth rates of evolved mutants and phenotypic restoration

To evaluate the forces of selection that operated during our evolutionary experiments, we quantified the growth rates of evolved mutants and compared them with their parental strains transformed by replicative plasmids bearing either *S. cerevisiae* or *Y. lipolytica* cognate RS genes (Supplementary Fig. 11). For the Asn-RS experiment, the presence of either the *macrotene* chromosome (BYAT580-345) or six episomal copies (BAT580-265) strongly accelerated generation times (124 and 148 min, respectively, compared with 353 min for BYAT583-0). There is, therefore, an enormous selective advantage for these mutants over their non-amplified parent (note that BYAT583-0 already had three episomal copies). The generation times of these mutants are even better than those of the transformants of BYAT580-0 by centromeric or multicopy replicative plasmids containing the *YALI* Asn-RS gene (167 and 152 min, respectively). For the Lys-RS experiment, the presence of either the *macrotene* chromosome (BYAT583-345) or the large episome (BYAT583-200) also reduced generation times significantly (192 and 183 min, respectively) compared with the non-amplified parental construct. In this case, the figure of 439 min observed for BYAT581-0 (an isogenic construct to BYAT583, Supplementary Table 1) is probably closer to the actual parental construct than the 284 min observed for BYAT583-0 that contained a segmental duplication (hence, three copies of the *YALI* Lys-RS gene). Phenotypic restoration in these evolved mutants was less efficient than the values obtained for transformants of BYAT581-0 or BYAT583-0 with the plasmids bearing Lys-RS gene.

In conclusion, compared with the great differences observed between evolved mutants and their parents, only minor phenotypic differences separated the low-order amplifications brought by episomes from the high-order amplifications of the *macrotene* chromosomes. In other words, the phenotypic advantage obtained by the first increments of *YALI*-RS gene copies was greater than the benefit obtained by further copy number increase. This is consistent with the moderate phenotypic differences observed in transformants (Supplementary Fig. 11). Multicopy plasmids reduced generation times by only 9–13% compared with centromeric plasmids bearing the *Y. lipolytica* genes. Growth curve data for strains of Supplementary Fig. 11 are available in Figshare http://figshare.com/s/6b33ab5a7af011e4bede06ec4bbcf141.

### Genetic bases of mutant phenotypes

Beside amplifications, we looked for the presence of SNPs and indels, which might have been accidentally selected during our evolutionary experiments, by careful analysis of genome sequence data (Methods). Only a few nucleotide substitutions occurred in our sequenced strains (no indel), some of which affecting CDS (Supplementary Table 2). Those common to all sequenced strains of a given evolutionary experiment and absent from the other (N01-N06 for the BYAT580 series and K01-K06 for the BYAT583 series) must represent mutational events occurred during strain construction that cannot determine phenotypic differences between evolved mutants and their parents. None of them falls within the amplicons described above. The other mutations fall in functionally diverse genes, making unlikely their specific selection in our experiments. All are in heterozygous form with the wild-type allele and may influence the phenotypes of evolved mutants if not recessive (not studied). Note that three mutations (N11, K01 and K14) affected genes involved in DNA replication stress response, a function that may be relevant to the phenomenon studied.

**Figure 5:**
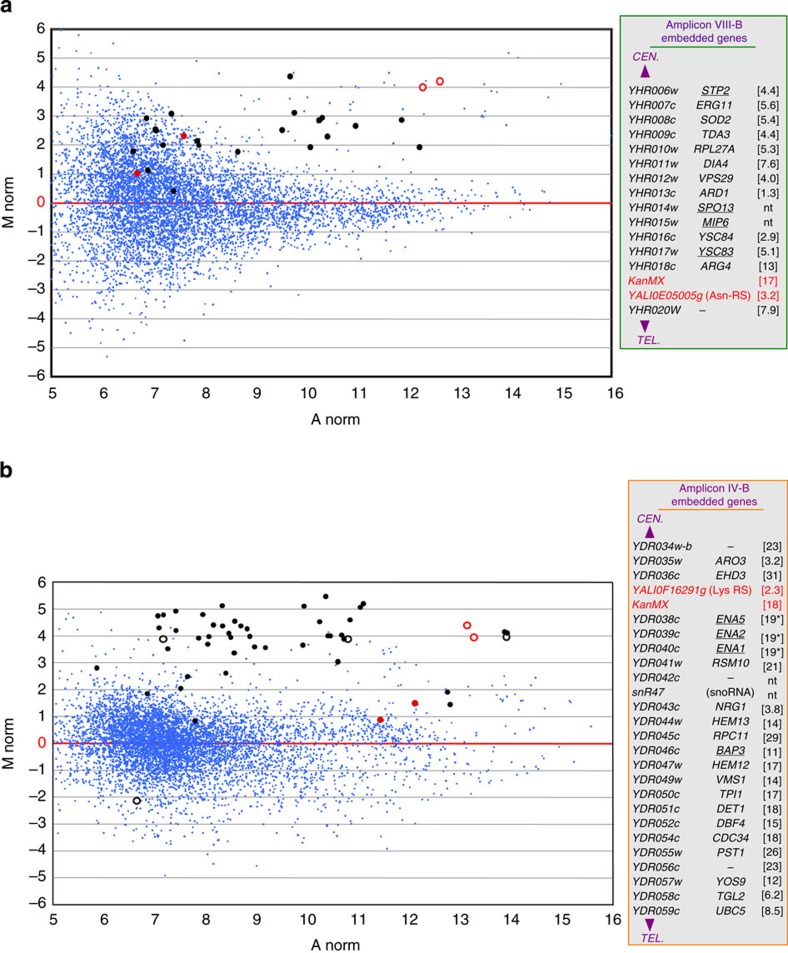
Transcriptome analysis of evolved strains bearing *macrotene* chromosomes. (**a**) Comparison of mutant BYAT580-345 with a non-amplified strain (BYAT521). MA plot log2 ratios of RNA levels (see Methods). Abscissae: transcript abundance (normalized log_2_), ordinates: log_2_ ratio of transcript abundance between the two compared strains. Black dots: amplicon-embedded *S. cerevisiae* genes (lists in right boxes with fold excess of RNA levels in BYAT580-345 under brackets. nt: not tested. Underlined: toxic gene when overexpressed^41^,^42^). The 14 amplicon-embedded genes (2 not tested) are overexpressed 6.2 times on average (standard deviation 4.2) in strain BYAT580-345 (14 amplicon copies) compared with strain BYAT521 (2 amplicon copies). Black circles: noncoding RNA genes. Red dots: *Y. lipolytica* RS genes. Red circles: KanMX. Small blue dots: all others. (**b**) Comparison of mutant BYAT583-345 with a non-amplified strain (BYAT581-0). Same legend as above. The 24 amplicon-embedded genes (2 not tested) are overexpressed 16.2 times on average (standard deviation 7.7) in strain BYAT583-345 (22-24 amplicon copies) compared with strain BYAT581-0 (2 amplicon copies).

The last important question to interpret observed phenotypes concerns expression of the amplicon-embedded genes. This was addressed by comparing transcriptomes of evolved mutants bearing the *macrotene* chromosomes with non-amplified strains (Fig. 5). We found that nearly all amplicon-embedded genes were overexpressed in strains carrying the *macrotene* chromosomes, with transcript levels roughly proportional to repeat numbers (6.2 times on average for the 14 genes of amplicon VIII-B in strain BYAT580-345 for 14 copies instead of 2, and 16.2 times on average for the 24 genes of amplicon IV-B in strain BYAT583-345 for 22-24 copies instead of 2). Overexpression factor varied between genes but independently from normal transcript abundance (a dynamic range of 50- to 100-fold was observed). The *YALI*-RS genes themselves were also overexpressed, although to a lesser extent possibly due to imperfect transcript stability in *S. cerevisiae* cells. The increase of transcript levels with gene copy numbers is, obviously, not surprising and often empirically used for heterologous gene expression. In yeast, genes along with disomic chromosomes in aneuploid strains are overexpressed^39^,^40^. But quantitative data were needed for the high-order amplifications of our *macrotene* chromosomes, in particular because some of these genes were known to be toxic to cells when overexpressed^41^,^42^. Normalized data for Fig. 5 and Supplementary Fig. 3 are available in GEO under the accession code GSE64431.

## Discussion

The *macrotene* chromosomes described here are remarkable by their combination of large repeat units (30–50 kb), embedding many genes, with high copy numbers (12–22) such as to form repeat arrays roughly half the sizes of chromosomes. This distinguishes them from most previously reported structures in yeast formed of duplications only when affecting large chromosomal segments^7^,^8^,^13^,^14^,^23^,^25^ or affecting short size segments only when amplified in multiple^32^,^33^ or low (4–5)^8^,^9^,^12^,^26^,^34^ copy numbers. The same balance looks true in other eukaryotes^20^,^22^,^29^,^30^, with only limited exceptions so far^43^,^44^. High-order tandem amplifications of large chromosomal segments, equivalent to those forming the yeast *macrotene* chromosomes, were reported long ago in the lactose operon of *E. coli*^45^ and were recently interpreted in terms of a stress-induced template switching mechanism during replication^46^. The recent example of massive amplification of large chromosomal segments in *Plasmodium falciparum* in response to continuous *in vivo* challenge with a novel inhibitor of the dihydro-orotate dehydrogenase^44^ represents, probably, the closest structure to our yeast *macrotene* chromosomes. In this case, however, amplifications resulted from a two-step selection process involving, first, the formation of classical segmental duplications (with junctions in the many A/T-rich tracts of this genome) and, second, their gradual head-to-tail expansion by a classical homology-based recombination mechanism between repeats driven by increasing levels of drug. No similar intermediates were found in our experiments, raising the question of the number and nature of mutational steps at the origin of the *macrotene* chromosomes. Evolved clones picked up at various stages of the evolutionary experiments (Tables 1 and 2) contained *macrotene* chromosomes indistinguishable in size from the sequenced ones, as if repeat numbers remained nearly invariable. An involuntary selection of larger colonies during mutant isolation is, of course, possible. But the limited phenotypic differences observed between mutants with high and low orders of amplification makes it unlikely. Mutants with intermediate amplifications should have been isolated if they ever existed.

In absence of such intermediates during the formation of *macrotene* chromosomes, this role could have been played by the circular episomes observed in both experiments, especially in chromosome VIII where the repeat junctions of amplicon VIII-B were identical to the junction of the episome (Supplementary Fig. 9) and where several mutants carrying both structures simultaneously appeared before the emergence of mutants with the *macrotene* chromosome only (Table 1). The reintegration of circular episomes into chromosomes, forming extensive tandem amplifications through a rolling-circle type of mechanism, was previously reported for small artificial plasmids^47^ and small telomeric circles^48^. In both cases, however, the tandem arrays were formed in telomeric position, not *in loco* as observed here. In addition, amplicon VIII-B units are not exact copies of the episome because of the 25-kb internal deletion of the pericentromeric region (Fig. 4a and Supplementary Fig. 9). In the case of chromosome IV, the early appearance of *macrotene* chromosome and the complete absence of coincidence between amplicon IV-B units and the latter appearing 211 kb episome also excludes this hypothesis. By contrast, the transient aneuploidy, observed twice, might be necessary for the appearance of this *macrotene* chromosome, albeit clearly not required for its maintenance (Supplementary Fig. 10).

These considerations, together with the limited phenotypic gain provided by additional copies of the *YALI*-RS genes beyond a small number, lead us to the hypothesis that the formation of *macrotene* chromosomes could have been direct, one-step events from the non-amplified parental genomes of the severely disabled strains, and not step-wise events of recombination between pre-existing intermediates driven by gradual fitness increase. The fact that only one chromosomal copy was affected each time, although strains were diploid, strengthens this idea, and suggests that an accidental mechanism transformed directly one amplicon unit into many within the affected chromosome, as would do a rolling-circle type of replication. Single-stranded DNA annealing during altered fork progression^49^ or re-initiation of replication origins^50^ could possibly create such an uncontrollable structure but it requires sequence homology, whereas the repeat junctions of our *macrotene* chromosomes involved distinct (VIII-B and right border of IV-B) or diverged sequences (internal VIII-B deletion and left border of IV-B). An alternative possibility would be accidental template switches during replication, a known stress-stimulated phenomenon^27^,^28^,^46^. Depending upon their location and orientation relative to replication origins, switches within or between adjacent replication forks have the intrinsic power to generate direct or inverted tandems, as observed (direct junction *YHLCdelta1*-*YHRCdelta3* in *macrotene* chromosome VIII or inverted junction *YDRCdelta6a*-*YDRWdelta7* in *macrotene* chromosome IV). The switches may involve nascent Okazaki fragments during perturbed replication fork progression in our severely unfit cells. Or they may be provoked by interference of short DNA or even RNA molecules whose actions in gene amplification and DNA repair were previously demonstrated in yeast^51^,^52^. The ‘guide’ sequence postulated for amplicon VIII-A circularization and amplicon VIII-B tandem junction may rely on such mechanisms, whereas the quasi-palindromic junction at the right of amplicon IV-B may witness accidental strand breakage. Additional experiments are obviously needed to precisely determine these mechanisms but, given their characteristics, *macrotene* chromosome amplifications are certainly worth considering for the evolution of eukaryotic genomes during normal or pathological cellular proliferation.

## Methods

### Cultures conditions

Yeast strains were grown on YPD medium (yeast extract 10 g l^−1^, bacto peptone 10 g l^−1^, glucose 20 g l^−1^, with or without 25 g l^−1^ bacto-agar as needed), at 30 °C unless otherwise indicated, or synthetic complete medium (yeast nitrogen base 6.7 g l^−1^, glucose 20 g l^−1^, plus all amino acids, uracil and adenine, with or without 25 g l^−1^ bacto-agar as needed).

### Yeast strains

All *S. cerevisiae* strains are S288c derivatives (Supplementary Table 1). Genetically disabled strains, used to initiate evolutionary experiments, were constructed by replacement of either the asparagine- or the lysine-tRNA synthetase gene by its orthologue from *Y. lipolytica*, as detailed in Supplementary Note 2. Diploid strains bearing homozygous gene replacement for the Asn-RS gene (BYAT580) or the Lys-RS gene (BYAT581 and BYAT583) were phenotypically unfit. Evolved strains derived from BYAT580 or BYAT583 in the primary evolutionary cultures were designated by the approximate total number of generations from inoculums at time of their isolation (Tables 1 and 2). Subclones of evolved mutants were isolated after 145 additional generations (see Supplementary Fig. 10) and designated as indicated in the text.

### Gene cloning and yeast transformations

Plasmids were propagated in *E. coli* strain XL1 blue (endA1 gyrA96(nalR) thi-1 recA1 relA1 lac glnV44 F'[::Tn10 pro AB^+^ lacIq Δ(lacZ)M15] hsdR17 (r_K_^−^ m_K_^+^)). Replacement of Asn-RS (*YHR019c*) and Lys-RS (*YDR037w*) genes of *S. cerevisiae* by their orthologues from *Y. lipolytica* (*YALI0E05005g* and *YALI0F16291g*, respectively) was done by transformation of the diploid strain BYAT290 and selection of transformants on YPD medium containing 200 μg ml^−1^ of G418. Transformations of strains BYAT580-0, BYAT583-0 and BYAT581-0 by replicative plasmids containing the Asn-RS or Lys-RS genes from *S. cerevisiae* or *Y. lipolytica* were done by *in vivo* homologous recombination after co-transformation of yeast cells by 500 ng of *Bam*HI-linearized plasmid DNA with 1 μg of a PCR-amplified genomic fragment bearing the RS gene of interest. Plasmids were pRS415 (ARS-based centromeric) and pRS425 (2 μm-based multicopy) *E.coli–S. cerevisiae* shuttle vectors containing the *LEU2* marker^53^. PCR amplifications of RS genes were made on total genomic DNA from the natural *S. cerevisiae* strain FY1679 (ref. 54) (for *SACE* RS genes), BYAT580-345 (for the *YALI* Asn-RS gene) or BYAT583-345 (for the *YALI* Lys-RS gene) using pairs of oligonucleotides with 18- to 22-nt-long 3' parts homologous to the promoter or terminator regions of the *S. cerevisiae* RS genes and 50-nt-long 5' parts homologous to left or right sides of the *Bam*HI sites of plasmids. Amplified segments extended from coordinate 141,406 to 143,938 on chromosome VIII and from coordinate 524,771 and 527,466 on chromosome IV. Transformants were selected on synthetic complete-Leu medium.

### Other basic techniques

Yeast tetrads were micromanipulated using Singer MSM equipment. Ascospores were inoculated on thin YPD agar medium and incubated at 30 °C for 3 days, following which the agar was placed on top of new YPD plates to ensure nutrient availability for slow growing strains, and further incubated for several days.

PFGEs were run in 1% agarose gels, 0.25 × Tris borate EDTA buffer at pH 8.3 at 12 °C and 5 V cm^−1^ for 65 h on Rotaphor (Biometra) with an alternating field angle of 120° and various pulse ramps (Fig. 3). Chromosomal DNA was prepared from agarose-embedded yeast cells according to standard methods.

Generation times of yeast strains were determined by optical density measurement in 150 μl liquid YPD cultures in paraffin-sealed 96-well microtitre plates using Sunrise microplate reader (TECAN) with rotational shaking. Each well was inoculated with ca 10^4^ freshly grown cells and cultures were incubated at 30 °C with automated optical density measurements at 620 nm every 10 min for 30 to 50 h. Average generation times (expressed in minutes) were calculated from optical density values (Supplementary Fig. 11).

### Evolutionary experiments

Parental strains were inoculated in YPD liquid medium and grown at 30 °C by serial transfers for a total of ca 200 successive generations (Fig. 1a). Each transfer was made in 2 l of fresh medium with inoculums of 10^9^ cells. Under such conditions, each culture represents 7.3–8.9 generations (Tables 1 and 2), and a mutant with increased growth rate was able to invade subsequent cultures as soon as it reached a frequency of 10^−9^ in the evolving population. Note that a mutant with a growth rate increase of only 12.5% nearly doubles its relative frequency at each transfer. After each culture, cells were counted, diluted and plated on YPD medium to monitor growth and morphology of resulting colonies (Fig. 1b). Faster growing mutants (larger colonies) were picked up for molecular analysis. Given this protocol, mutants isolated from the successive cultures of a same evolutionary experiment (‘evolved strains’) may or may not derive from the same mutational event(s).

### Deep sequencing analysis

Shotgun libraries were prepared by standard Illumina protocols using 5 μg (GAIIx) or 1 μg (HiSeq2000) of total genomic DNA from selected strains, and sequenced at different depths (23–43 X for GAIIx and 170-440 X for HiSeq2000). Sequence data were analysed according to the pipeline illustrated by Supplementary Fig. 12. All reads were first submitted to FastQC v0.10.1 Babraham Bioinformatocs ( http://www.bioinformatics.babraham.ac.uk/projects/fastqc/) and regions of problematic base calling (deviation from Chargaff's rules) were trimmed off using Fastx Toolkit v0.0.13 (http://hannonlab.cshl.edu/fastx_toolkit/). Trimmed reads were then aligned along the 16 chromosomes of *S. cerevisiae* S288c (GenBank NC_001133 to NC_001148, PLN 06-DEC-2008), plus the 2 RS genes of *Y. lipolytica* (*YALI0E05005g* and *YALI0F16291g*) using single-end mapping mode of BWA v0.6.2 (ref. 55) with default parameters. Output SAM files were converted to BAM files using SAMtools v0.1.18 (ref. 56). For each yeast strain, sequencing coverage along chromosome maps was computed by BEDtools v2.17.0 5 (ref. 57). Values were normalized to 2 (for diploid number) from mean coverage over entire genome. When necessary, curves were smoothed using 1,500 nt (Fig. 2) or 5,000 nt slinding windows (Supplementary Fig. 5). Unmapped reads were reassembled using Spades 3.0.0 (ref. 58) (k-mer size of 17) to detect possible breakpoints in evolved strains. All unmapped reads were also aligned against *Y. lipolytica* tRNA genes using the single-end mapping mode of BWA v0.6.2 with default parameters to ascertain the absence of such genes in the genomes of parental strains and evolved mutants.

SNPs and indels were identified from BAM alignment files processed using SAMtools v0.1.18 (ref. 56), GATK v2.2 (ref. 59) and Picard v1.81 ( http://picard.sourceforge.net/). The ‘Add Read Groups’ step was made by Picard. Aligned reads were realigned with the command IndelRealigner from GATK. Duplicated reads were removed by MarkDuplicates, implemented in Picard. We only kept reads that were uniquely mapped to the reference sequence by SAMtools. Positions corresponding to repeated regions such as telomeric repeats, Y', Ty elements, LTR, *MAT*, *HML* and *HMR* loci, rDNA and *CUP1* loci, totalling 3.8% of the genome, were filtered out. SAMtools was used to generate *mpileup* files without BAQ adjustments. SNP and Indel calls were processed by Varscan v2.3.2 (ref. 60). The *mpileup* files were used to call the mismatches (SNPs and indels) with the options ‘mpileup2snp’ and ‘mpileup2indel’ of Varscan2 with a minimum depth of 5 reads at the position to make a call and a threshold of 0.2 for minimum variant allele frequency (strains are diploids). In amplicon regions, mismatches were called as above, except that no minimum read depth and no minimum variant allele frequency were considered.

### Point mutation detection

Candidates for nucleotide substitutions and indels identified from above pipeline were compared between all sequenced strains. Common candidates to all strains were considered as errors in the S288c reference sequence used (above) when they appeared in homozygous form (majority), or to mutational events occurred during initial strain construction steps when appearing in heterozygous form. They were subsequently ignored. Remaining candidates were manually verified. Sequence reads were reexamined using *Tablet*^61^, checking consistency between reads of opposite orientations (to eliminate PCR amplification bias) and alignments (to eliminate erroneous indels). In final, a total of 29 non-synonymous or non-sense mutational changes and 8 synonymous changes were found in annotated CDS (Supplementary Table 2), plus roughly a third of this total number falling in intergenic regions as expected from the *S. cerevisiae* genome annotation. No indel candidate was validated after manual verification.

### Transcriptome analysis

Total RNAs extracted from exponentially growing cells on YPD medium were reversed transcribed with Cy3 or Cy5 labels (InVitroGen Superscript indirect cDNA Labeling System) and hybridized against custom-designed Agilent microarrays of 50-mer synthetic oligonucleotides (AMADID 050530). Each array contained triplicates of 5,940 and 5,660 probes specific, respectively, for upstream and downstream parts of annotated *S. cerevisiae* CDS (dubious CDS ignored), as well as 4 probes for the *Y. lipolytica* Asn-RS and Lys-RS genes (upstream and downstream parts of *YALI0E05005g* and *YALI0F16291g*, respectively) and 2 probes for the *Kan*MX marker. The arrays also contained triplicates of 427 probes for *S. cerevisiae* spliceosomal introns, 1,233 probes for Cryptic Unstable Transcripts (CUTs)^62^, 1,304 probes for other noncoding RNAs and 23 probes for mitochondrial DNA. Comparative hybridizations (with dye swap) were scanned on AXON GENEPIX 4200AL scanner. Data were analysed using GenePix Pro 7 (Molecular Devices). Data drawings represent MA plot log2 ratios (R ‘arrays’ package Bioconductor R:2.13(3.0.1), http://www.bioconductor.org).

## Author contributions

A.T. performed all experimental work and contributed to sequence data interpretation. V.K. established a sequence data analysis pipeline and contributed to interpretation. S.C. formatted the original sequence data. I.L. contributed to early sequence data analysis. L.M. performed all sequencing on Illumina sequencers. C.B. coordinated the sequencing platform. B.D. conceived and coordinated the project and wrote the paper.

## Additional information

**Accession codes.** DNA sequencing data for BYAT580-0, BYAT580-60, BYAT580-120, BYAT580-200, BYAT580-265 and BYAT580-345, and for BYAT583-0, BYAT583-60, BYAT583-120, BYAT583-200 and BYAT583-345 have been deposited in the European Nucleotide Archive http://www.ebi.ac.uk/ena/data/view/ERP008906 under the accession codes ERS622562 to ERS622572, respectively.

**How to cite this article:** Thierry, A. *et al.* *Macrotene* chromosomes provide insights to a new mechanism of high-order gene amplification in eukaryotes. *Nat. Commun.* 6:6154 doi: 10.1038/ncomms7154 (2015).

## Supplementary Material

Supplementary Information Supplementary Figures 1-12, Supplementary Tables 1-3, Supplementary Notes 1-3 and Supplementary References.

## Figures and Tables

**Table 1:** Evolutionary trajectory of the BYAT580 population.

**Culture #**	**Culture duration (days)**	**Final culture density per ml ( × 10**^8^**)**	**No. of generations in the culture (*****n*****)**	**Total no. of generations (cumul)**	**GEN.**	**First evolved mutant isolated**	**Second evolved mutant isolated**
**1**	**3**	**1.2**	**11.2**	**11.2**	**0**	**BYAT580-0 [E] a, b, c**	
2	7	1.2	8.9	20.1			
3	4	1.0	7.6	27.8			
4	2	1.8	8.5	36.3			
5	6	1.7	8.4	44.7			
**6**	**3**	**2.0**	**8.6**	**53.3**	**60**	**BYAT580-60 [E] a, b, c**	BYAT580-61 [E] b
7	3	1.9	8.6	61.9	70	BYAT580-70 [E] a, b	BYAT580-71 [E] b
8	2	1.8	8.5	70.4	80	BYAT580-80 [E] a, b	BYAT580-81 [E] b
9	2	1.7	8.4	78.8	90	BYAT580-90 [E] a, b	
10	3	1.7	8.4	87.2	100	BYAT580-100 [E] a, b	BYAT580-101 [E] b
11	2	2.0	8.6	95.8	110	BYAT580-110 [E] a, b	
12	2	1.5	8.2	104.1			
**13**	**5**	**1.6**	**8.3**	**112.4**	**120**	**BYAT580-120 [E] a, b, c**	BYAT580-121 [E] a, b
14	2	1.7	8.4	120.8	130	BYAT580-130 [E] a, b	
15	3	1.5	8.2	129.0	140	BYAT580-140 [M] a, b	BYAT580-141 [E] a, b
16	2	2.0	8.6	137.7	150	BYAT580-150 [E] a, b	BYAT580-151 [E+M]* a, b
17	2	1.5	8.2	145.9			
18	3	1.6	8.3	154.2			
19	4	1.8	8.5	162.7	170	BYAT580-170 [E+M] a, b	BYAT580-171 [E+M] a, b
20	3	1.9	8.6	171.3			
21	4	1.9	8.6	179.9	190	BYAT580-190 [E] a, b	BYAT580-191 [E] a, b
22	10	2.3	8.8	188.7			
**23**	**10**	**2.4**	**8.9**	**197.6**	**200**	**BYAT580-200 [M] a, b, c**	BYAT580-201 [E+M]* a, b

Left part: Details of the serial-transfer cultures. At the end of each 2-l culture in YPD medium, final cell density (*d*, column 3) was estimated by Malassez cell counting (on diluted aliquots), and the number of generations of the culture (*n*, column 4) was calculated from the final cell number (Nf=2,000 × *d*) relative to the inoculum (Ni) according to the equation *n*=(ln (Nf)-ln (Ni))/ln (2). Each inoculum (Ni) was made of 10^9^ cells from the previous culture, except for cultures #1 and 2 (10^8^ and 5 × 10^8^ cells, respectively). Right part: Cultures were designated by approximate generation numbers (GEN.) throughout this work (note the slight differences from actual numbers, left part column 5). Last two columns list subclones picked up from evolving populations (designated according to GEN.) with their genomic structures under brackets ([E]: circular episome, [M]: macrotene chromosome, *altered chromosome I), followed by the experimental evidence(s) supporting the structures (a: PFGE probed with the *YALI* Asn-RS gene, see Fig. 3; b: *Nsi*I digest probed with *YALI* Asn-RS gene; c: *Bam*HI digest probed with *YHR020w,* see Supplementary Fig. 7). Bold entries are sequenced genomes.

**Table 2:** Evolutionary trajectory of the BYAT583 population.

**Culture #**	**Culture duration (days)**	**Final culture density per ml ( × 10**^8^**)**	**No. of generations in the culture (*****n*****)**	**Total no. of generations (cumul)**	**GEN.**	**First evolved mutant isolated**	**Second evolved mutant(s) isolated**
**1**	**3**	**1.2**	**11.2**	**11.2**	**0**	**BYAT583-0 [SD] a, d, e, f, g**	
2	7	0.9	8.5	19.7			
3	4	1.6	8.3	28.0			
4	2	1.4	8.1	36.2			
5	6	1.1	7.8	44.0			
**6**	**3**	**1.6**	**8.3**	**52.3**	**60**	**BYAT583-60 [M+SD] a, d, e, f, g**	
7	3	1.4	8.1	60.4	70	BYAT583-70 [M] a	
8	2	1.4	8.1	68.5	80	BYAT583-80 [M] a, d	BYAT583-81 [M] d
9	2	1.3	8.0	76.6	90	BYAT583-90 [M] a, d	
10	3	1.1	7.8	84.3	100	BYAT583-100 [M] a, d	
11	2	1.3	8.0	92.4	110	BYAT583-110 [M] a, d	BYAT583-111 [M] d
12	2	1.1	7.8	100.1			
**13**	**5**	**1.4**	**8.1**	**108.3**	**120**	**BYAT583-120 [M+SD] a, d, e, f**	BYAT583-121 [M] a
14	2	1.1	7.8	116.1	130	BYAT583-130 [M] a	
15	3	1.0	7.6	123.7	140	BYAT583-140 [E] a, d	BYAT583-141 [M] d
16	2	1.6	8.3	132.0	150	BYAT583-150 [E] a, d	BYAT583-151 [E] d
17	2	1.2	7.9	139.9			
18	3	1.3	8.0	147.9			
19	4	1.8	8.5	156.4	170	BYAT583-170 [E+SD] a	
20	3	1.8	8.5	164.9			
21	4	1.6	8.3	173.3	190	BYAT583-190 [E+SD] a,d	BYAT583-191 [E] d
22	10	1.8	8.5	181.7			
**23**	**10**	**2.1**	**8.7**	**190.5**	**200**	**BYAT583-200 [E+SD] a, d, e, f, g**	BYAT583-201 [E] d BYAT583-202 [E] d

Left part: Same explanation as in Table 1. Right part: Same explanation as in Table 1 except for an additional structure: [SD] segmental duplication of amplicon IV-A, and for experimental evidence(s): (a: PFGE probed with the *YALI* Lys-RS gene, see Fig. 3; d: *Xho*I digest probed with *YALI* Lys-RS gene; e: *Eco*RV digest probed with *YDR035c*; f: *Bam*HI digest probed with *YDR098c*; g: *Eco*RV digest probed with *YDR098c*; see Supplementary Fig. 8).

## References

[b1] Dettman J. R. *et al.* Evolutionary insight from whole-genome sequencing of experimentally evolved microbes. Mol. Ecol. 21, 2058–2077 (2012). 22332770 10.1111/j.1365-294X.2012.05484.x

[b2] Yates L. R. & Campbell P. J. Evolution of cancer genome. Nat. Rev. Genet. 13, 795–806 (2012). 23044827 10.1038/nrg3317 PMC3666082

[b3] Adey A. *et al.* The haplotype-resolved genome and epigenome of the aneuploid HeLa cancer cell line. Nature 500, 207–211 (2013). 23925245 10.1038/nature12064 PMC3740412

[b4] Barrick J. E. *et al.* Genome evolution and adaptation in a long-term experiment with *Escherichia coli*. Nature 461, 1243–1247 (2009). 19838166 10.1038/nature08480

[b5] Lynch M. *et al.* A genome-wide view of the spectrum of spontaneous mutations in yeast. Proc. Natl Acad. Sci. USA 105, 9272–9277 (2008). 18583475 10.1073/pnas.0803466105 PMC2453693

[b6] Serero A. *et al.* Mutational landscape of yeast mutator strains. Proc. Natl Acad. Sci. USA 111, 1897–1902 (2014). 24449905 10.1073/pnas.1314423111 PMC3918763

[b7] Dunham M. J. *et al.* Characteristic genome rearrangements in experimental evolution of *Saccharomyces cerevisiae*. Proc. Natl Acad. Sc. USA 99, 16144–16149 (2002). 12446845 10.1073/pnas.242624799 PMC138579

[b8] Gresham D. *et al.* The repertoire and dynamics of evolutionary adaptations to controlled nutrient-limited environments in yeast. PLoS Genet. 4, e1000303 (2008). 19079573 10.1371/journal.pgen.1000303 PMC2586090

[b9] Araya C. L. *et al.* Whole-genome sequencing of a laboratory-evolved yeast strain. BMC Genomics 11, 88 (2010). 20128923 10.1186/1471-2164-11-88 PMC2829512

[b10] Gresham D. *et al.* Adaptation to diverse nitrogen-limited environments by deletion or extrachromosomal element formation of the *GAP1* locus. Proc. Natl Acad. Sc 107, 18551–18556 (2010). 20937885 10.1073/pnas.1014023107 PMC2972935

[b11] Dorsey M. *et al.* Spontaneous amplification of the *ADH4* gene in *Saccharomyces cerevisiae*. Genetics 132, 943–950 (1992). 1459445 10.1093/genetics/132.4.943 PMC1205250

[b12] Payen C. *et al.* The dynamics of diverse segmental amplifications in populations of *Saccharomyces cerevisiae* adapting to strong selection. G3 4, 399–409 (2014). 24368781 10.1534/g3.113.009365 PMC3962480

[b13] Koszul R., Caburet S., Dujon B. & Fischer G. Eukaryotic genome evolution through the spontaneous duplication of large chromosomal segments. EMBO J. 23, 234–243 (2004). 14685272 10.1038/sj.emboj.7600024 PMC1271662

[b14] Payen C., Koszul R., Dujon B. & Fischer G. Segmental duplications arise from *pol32*-dependent repair of broken forks through two alternative replication-based mechanisms. PLoS Genet. 4, e1000175 (2008). 18773114 10.1371/journal.pgen.1000175 PMC2518615

[b15] Libuda D. E. & Winston F. Amplification of histone genes by circular chromosome formation in *Saccharomyces cerevisiae*. Nature 443, 1003–1007 (2006). 17066037 10.1038/nature05205 PMC3365550

[b16] Huang T. & Campbell J. Amplification of a circular episome carrying an inverted repeat of the *DFR1* locus and adjacent autonomously replicating sequence element of *Saccharomyces cerevisiae*. J. Biol. Chem. 270, 9607–9614 (1995). 7721892 10.1074/jbc.270.16.9607

[b17] Walton J. D., Paquin C. E., Kaneko K. & Williamson V. M. Resistance to antimycine A in yeast by amplification of *ADH4* on a linear, 42 kb palindromic plasmid. Cell 46, 857–863 (1986). 3019553 10.1016/0092-8674(86)90067-x

[b18] Koszul R., Dujon B. & Fischer G. Stability of large segmental duplications in the yeast genome. Genetics 172, 2211–2222 (2006). 16489235 10.1534/genetics.105.048058 PMC1456401

[b19] Koszul R. & Fischer G. A prominent role for segmental duplications in modeling eukaryotic genomes. C R Biol. 332, 254–266 (2009). 19281956 10.1016/j.crvi.2008.07.005

[b20] Marques-Bonet T., Girirajan S. & Eichler E. E. The origins and impact of primate segmental duplications. Trends Genet. 25, 443–454 (2009). 19796838 10.1016/j.tig.2009.08.002 PMC2847396

[b21] Dujon B. Yeast evolutionary genomics. Nat. Rev. Genet. 11, 512–524 (2010). 20559329 10.1038/nrg2811

[b22] Chow E. W.L., Morrow C. A., Djordjevic J. T., Wood I. A. & Fraser J. A. Microevolution of *Cryptococcus neoformans* driven by massive tandem gene amplification. Mol. Biol. Evol. 29, 1987–2000 (2012). 22334577 10.1093/molbev/mss066

[b23] Hughes T. R. *et al.* Widespread aneuploidy revealed by DNA microarray expression profiling. Nature Genet. 25, 333–337 (2000). 10888885 10.1038/77116

[b24] Yona A. H. *et al.* Chromosomal duplication is a transient evolutionary solution to stress. Proc. Natl Acad. Sci. USA 109, 21010–21015 (2012). 23197825 10.1073/pnas.1211150109 PMC3529009

[b25] Zhang H. *et al.* Gene copy-number variation in haploid and diploid strains of the yeast *Saccharomyces cerevisiae*. Genetics 193, 785–801 (2013). 23307895 10.1534/genetics.112.146522 PMC3583998

[b26] Brewer B. J. *et al.* Origin-dependent inverted-repeat amplification: a replication-based model for generating palindromic amplicons. PLoS Genet. 7, e1002016 (2011). 21437266 10.1371/journal.pgen.1002016 PMC3060070

[b27] Lambert S. & Carr A. M. Replication stress and genome rearrangements: lessons from yeast models. Curr. Opin. Genet. Dev. 23, 132–139 (2013). 23267817 10.1016/j.gde.2012.11.009

[b28] Green B., Finn K. J. & Li J. L. Loss of DNA replication control is a potent inducer of gene amplification. Science 329, 943–946 (2010). 20724634 10.1126/science.1190966 PMC3700424

[b29] Arlt M. F. *et al.* Hydroxyurea induces *de novo* copy number variants in human cells. Proc. Natl Acad. Sci. USA 108, 17360–17365 (2011). 21987784 10.1073/pnas.1109272108 PMC3198378

[b30] Alt F. W., Kellems R. E., Bertino J. R. & Schimke R. T. Selective multiplication of dihydrofolate reductase genes in methotrexate-resistant variants of cultured murine cell. J. Biol. Chem. 253, 1357–1370 (1978). 627542

[b31] Kedes L. H. & Birnstiel M. L. Reiteration and clustering of DNA sequences complementary to histone messenger RNA. Nature New Biol. 230, 165–169 (1971). 5279989 10.1038/newbio230165a0

[b32] Welch J. W., Fogel S., Cathala G. & Karin M. Industrial yeasts display tandem gene iteration at the *CUP1* region. Mol. Cell. Biol. 3, 1353–1361 (1983). 6621529 10.1128/mcb.3.8.1353-1361.1983 PMC369981

[b33] Karin M. *et al.* Primary structure and transcription of an amplified genetic locus: The *CUP1* locus of yeast. Proc. Natl Acad. Sci. USA 81, 337–341 (1984). 6364141 10.1073/pnas.81.2.337 PMC344671

[b34] Brown C. J., Todd K. M. & Rosenzweig R. F. Multiple duplications of yeast hexose transport genes in response to selection in a glucose-limited environment. Mol. Biol. Evol. 15, 931–942 (1998). 9718721 10.1093/oxfordjournals.molbev.a026009

[b35] Dujon B. Yeasts illustrate the molecular mechanisms of eukaryotic genome evolution. Trends Genet. 22, 375–387 (2006). 16730849 10.1016/j.tig.2006.05.007

[b36] Dujon B. *et al.* Genome evolution in yeasts. Nature 430, 35–44 (2004). 15229592 10.1038/nature02579

[b37] Karakoc E. *et al.* Detection of structural variants and indels within exome data. Nat. Methods 9, 176–180 (2012). 22179552 10.1038/nmeth.1810 PMC3269549

[b38] Ho C. H. *et al.* A molecular barcoded yeast ORF library enables mode-of-action analysis of bioactive compounds. Nature Biotechnol. 27, 369–377 (2009). 19349972 10.1038/nbt.1534 PMC3856559

[b39] Torres E. M. *et al.* Effects of aneuploidy on cellular physiology and cell division in haploid yeast. Science 317, 916–924 (2007). 17702937 10.1126/science.1142210

[b40] Pavelka N. *et al.* Aneuploidy confers quantitative proteome changes and phenotypic variation in budding yeast. Nature 468, 321–325 (2010). 20962780 10.1038/nature09529 PMC2978756

[b41] Boyer J. *et al.* Large-scale exploration of growth inhibition caused by overexpression of genomic fragments in *Saccharomyces cerevisiae*. Genome Biol. 5, R72 (2004). 15345056 10.1186/gb-2004-5-9-r72 PMC522879

[b42] Sopko *et al.* Mapping pathways and phenotypes by systematic gene overexpression. Mol. Cell 21, 319–330 (2006). 16455487 10.1016/j.molcel.2005.12.011

[b43] Cook D. E. *et al.* Copy number variation of multiple genes at *Rhg1* mediates nematode resistance in soybean. Science 338, 1206–1209 (2012). 23065905 10.1126/science.1228746

[b44] Guler J. L. *et al.* Asexual populations of the human malaria parasite, *Plasmodium falciparum,* use a two-step genomic strategy to acquire accurate, beneficial DNA amplifications. PLoS Pathogens 9, e1003375 (2013). 23717205 10.1371/journal.ppat.1003375 PMC3662640

[b45] Tlsty T. D., Albertini A. & Miller J. H. Gene amplification in the *lac* region of *E. coli*. Cell 37, 217–224 (1984). 6327052 10.1016/0092-8674(84)90317-9

[b46] Lin D. *et al.* Global chromosomal structural instability in a subpopulation of starving *Escherichia coli* cells. PLoS Genet. 7, e1002223 (2011). 21901104 10.1371/journal.pgen.1002223 PMC3161906

[b47] Natarajan S., Groff-Vindman C. & McEachern M. J. Factors influencing the recombinational expansion and spread of telomeric tandem arrays in *Kluyeromyces lactis*. Euk. Cell 2, 1115–1127 (2003). 10.1128/EC.2.5.1115-1127.2003 PMC219379 14555494

[b48] Nosek J. *et al.* Amplification of telomeric arrays via rolling-circle mechanisms. J. Biol. Chem. 280, 10840–10845 (2005). 15657051 10.1074/jbc.M409295200

[b49] Feng W., Di Rienzi S. C., Raghuraman M. K. & Brewer B. J. Replication stress-induced chromosome breakage is correlated with replication fork progression and is preceded by single-stranded DNA formation. G3 1, 327–335 (2011). 22384343 10.1534/g3.111.000554 PMC3276152

[b50] Finn K. J. & Li J. J. Single-stranded annealing induced by re-initiation of replication origins provides a novel and efficient mechanism for generating copy number expansion via non-allelic homologous recombination. PLoS Genet. 9, e1003192 (2013). 23300490 10.1371/journal.pgen.1003192 PMC3536649

[b51] Mukherjee K. & Storici F. A mechanism of gene amplification driven by small DNA fragments. PLoS Genet. 8, e1003119 (2012). 23271978 10.1371/journal.pgen.1003119 PMC3521702

[b52] Storici F. *et al.* RNA-templated DNA repair. Nature 447, 338–341 (2007). 17429354 10.1038/nature05720 PMC2121219

[b53] Christianson T. W. *et al.* Multifunctional yeast high-copy number shuttle vectors. Gene 110, 119–122 (1992). 1544568 10.1016/0378-1119(92)90454-w

[b54] Engel SR. *et al.* The reference genome sequence of *Saccharomyces cerevisiae*: then and now. G3 4, 389–398 (2014). 24374639 10.1534/g3.113.008995 PMC3962479

[b55] Li H. & Durbin R. Fast and accurate short read alignment with Burrows-Wheeler transform. Bioinformatics 25, 589–595 (2009). 10.1093/bioinformatics/btp324 PMC2705234 19451168

[b56] Li H. *et al.* The Sequence alignment/map (SAM) format and SAMtools. Bioinformatics 25, 2078–2079 (2009). 19505943 10.1093/bioinformatics/btp352 PMC2723002

[b57] Quinlan A. R. & Hall I. M. BEDTools: a flexible suite of utilities for comparing genomic features. Bioinformatics 26, 841–842 (2010). 20110278 10.1093/bioinformatics/btq033 PMC2832824

[b58] Nurk S. *et al.* Assembling single-cell genomes and mini-metagenomes from chimeric MDA products. J. Comput. Biol. 20, 714–737 (2013). 24093227 10.1089/cmb.2013.0084 PMC3791033

[b59] DePristo M. *et al.* A framework for variation discovery and genotyping using next-generation DNA sequencing data. Nature Genet. 43, 491–498 (2011). 21478889 10.1038/ng.806 PMC3083463

[b60] Koboldt D. *et al.* VarScan 2: Somatic mutation and copy number alteration discovery in cancer by exome sequencing. Genome Res. 22, 568–576 (2012). 22300766 10.1101/gr.129684.111 PMC3290792

[b61] Milne I. *et al.* Tablet next generation sequence assembly visualization. Bioinformatics 26, 401–402 (2010). 19965881 10.1093/bioinformatics/btp666 PMC2815658

[b62] Neil H. *et al.* Widespread bidirectional promoters are the major source of cryptic transcripts in yeast. Nature 457, 1038–1042 (2009). 19169244 10.1038/nature07747

[b63] Berverley S. M. Characterization of the "unusual" mobility of large circular DNAs in pulsed field-gradient electrophoresis. Nucleic Acids Res 16, 925–939 (1988). 3344223 10.1093/nar/16.3.925 PMC334728

[b64] Nieduszynski C. A., Hiraga S.-I., Ak P., Benham C. J. & Donaldson A. D. OriDB: a DNA replication origin database. Nucleic Acids Res 35, D40–D46 (2007). 17065467 10.1093/nar/gkl758 PMC1781122

