# Supplementary material for: Macrotene chromosomes provide insights to a new mechanism of high-order gene amplification in eukaryotes
Source: Nat Commun. 2015 Jan 30;6:6154. doi: 10.1038/ncomms7154 (PMC4317496; doi:10.1038/ncomms7154)
Supplement: Supplementary Information — Supplementary Figures 1-12, Supplementary Tables 1-3, Supplementary Notes 1-3 and Supplementary References. [file ncomms7154-s1.pdf]

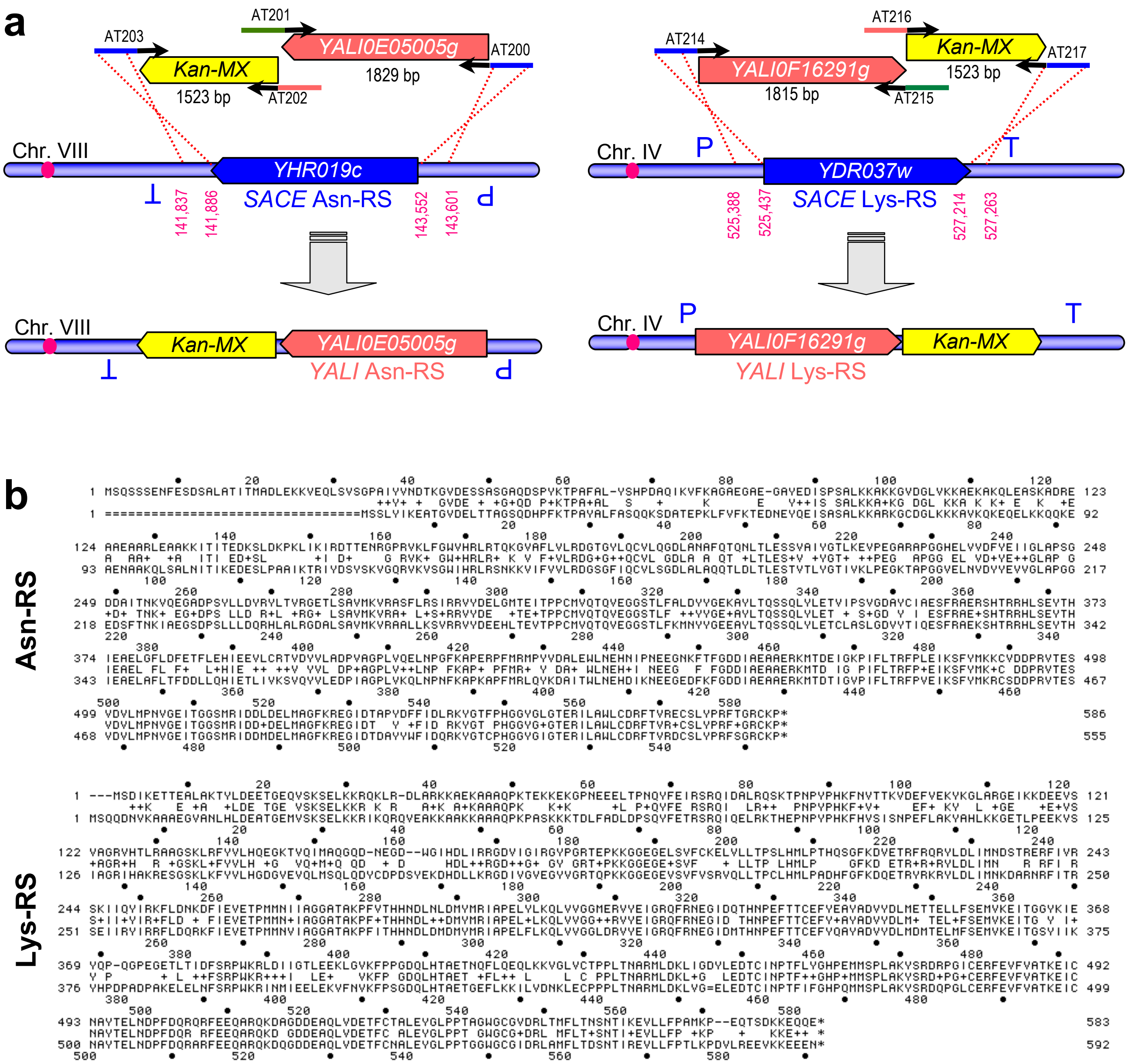


**Supplementary Figure 1: Orthologous replacement of tRNA synthetase genes and sequence divergence.**

**a.** Coding sequences of *S. cerevisiae* (*SACE*) Asn-RS or Lys-RS genes (blue) were precisely replaced by coding sequences of their *Y. lipolytica* (*YALI)* orthologs (pink), leaving intact promoter (P) and terminator (T) sequences, by homologous recombination within indicated sequence coordinates (dotted red lines). Transforming DNA cassettes were constructed by nested PCR amplifications, using synthetic oligonucleotides with 50 or 20 nt long 5' extensions (colored arrows) as primers (Atnnn, Supplementary Table 3). The *KanMX* gene (yellow) was used for the selection of integrative transformants. *Y. lipolytica* genes were amplified from DNA of sequenced strain CLIB99 [1]. PCR products and resulting transformants were sequenced to ensure absence of mutations. Note that a T > C transition occurred at position 1070 during amplification of *YALI0F16291g*, changing a UAU codon to its synonym UAC (Tyr).

**b.** Needleman-Wunsh sequence alignments (BLOSSUM 62 matrix, mismatch and gap penalties of 1 and 2 [2]) of cytoplasmic Asn-RS and Lys-RS enzymes of *Y. lipolytica* (top lines*)* and *S. cerevisiae* (bottom lines). Note the 33-aa long N terminal extension of the *YALI* Asn-RS. The two Asn-RS show 66.7 % amino-acid sequence identity over their shared length, compared to 67.5 % for the two Lys-RS.

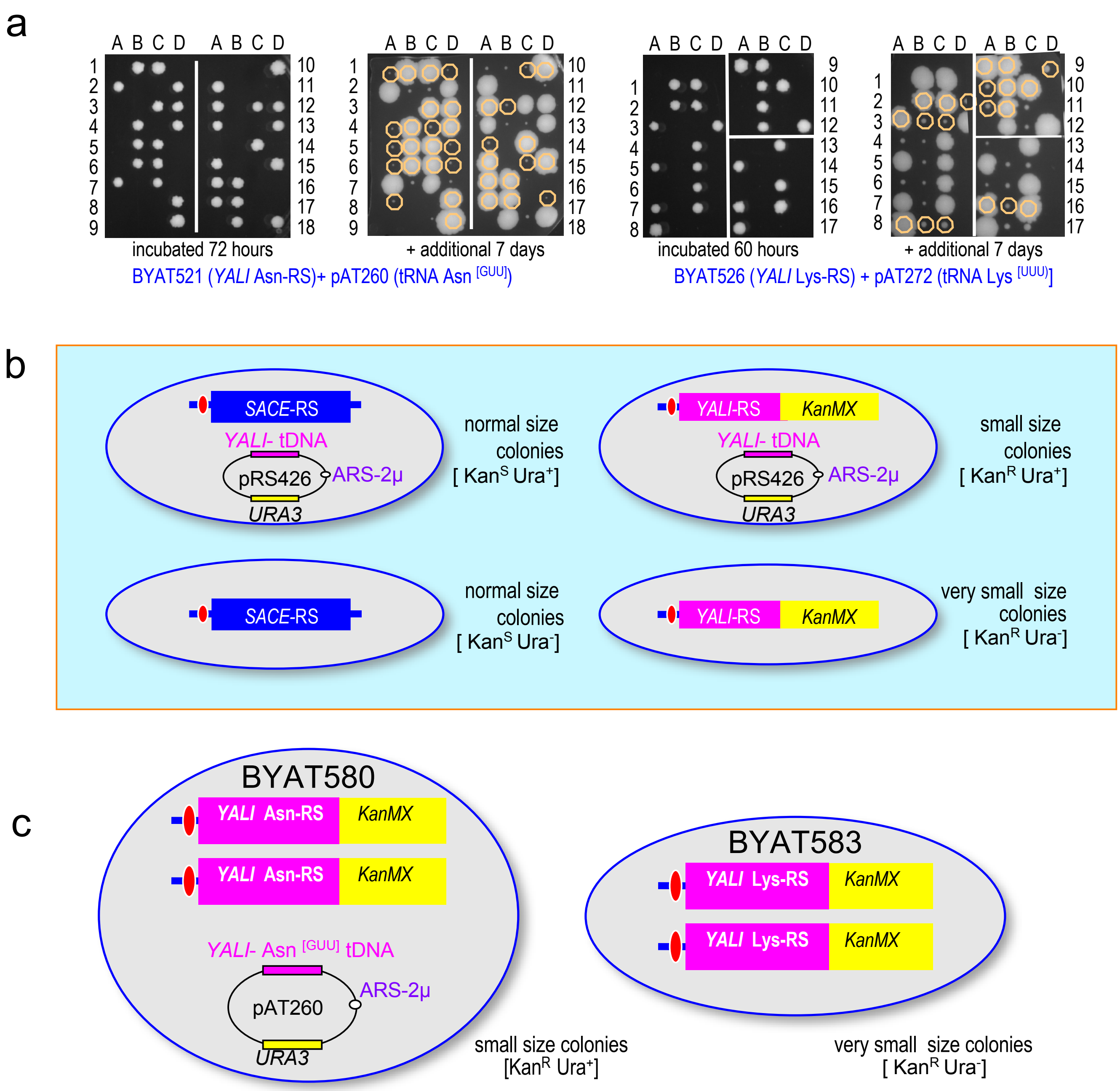


**Supplementary Figure 2: Construction of transgenic *S. cerevisiae* strains with *Y. lipolytica* Asn-RS or Lys-RS genes.**

**a.** Tetrad analysis of heterozygous *YALI*-RS / *SACE*-RS diploid strains containing multicopy plasmids with cognate *Y. lipolytica*-tDNAs (see Supplementary Table 1). Micromanipulated ascospores were incubated on YPD medium at 30°C for indicated periods of time. Note colony size differences. Ascospores bearing the *YALI* RS genes {Kan$^R$] make visible colonies only after 7 additional days of incubation on fresh YPD medium. Few complete tetrads were obtained for BYAT526 (mostly triads). Colonies marked by a circle are phenotypically Ura$^+$, indicating the presence of a plasmid.

**b.** Cartoons summarizing the genetic make up of the four types of meiotic segregants from BYAT521 and BYAT526, with indication of phenotypes (right). pRS426 is a 2 µ-based *E.coli-S.cerevisiae* shuttle vector [3].

**c.** Same cartoons for final homozygous diploid strains, BYAT580 and BYAT583, used for the evolutionary experiments. pAT260 is a derivative of pRS426 bearing the YALI Asn [GUU] tDNA (see Supplementary Table 1).

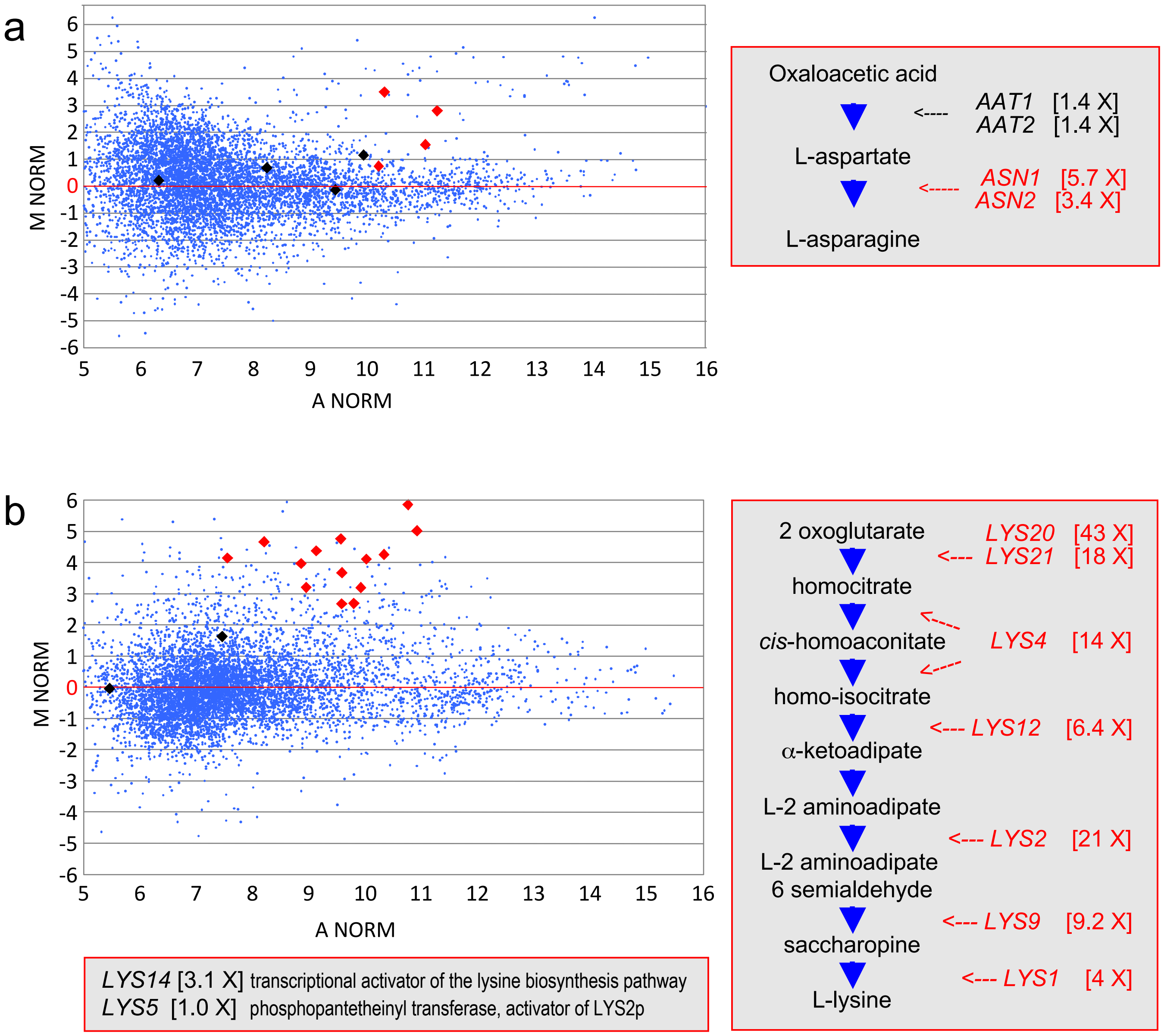


**Supplementary Figure 3: Microarray-based transcriptome analysis of transgenic strains.**
**a.** MA plot log2 ratios of RNA levels of BYAT580-0 (*YALI* Asn-RS / *YALI* Asn-RS) compared to BYAT521 (*YALI* Asn-RS / *SACE Asn-RS)*. Same legend as Fig. 5. Red dots: *ASN1* and *ASN2* genes. Black dots: *AAT1* and A*AT2 genes.* Right box: asparaginyl biosynthetic pathway and corresponding genes. Fold excess of RNA levels computed from mean M NORM are under brackets. Note that the phenotype of BYAT580-0 was already partly restored (Supplementary Fig. 10) owing to the presence of 3 copies of amplicon VIII-A (episome) in excess to diploid number (Fig. 2).
**b.** MA plot log2 ratios of RNA levels of BYAT581-0 (*YALI* Lys-RS / *YALI* Lys-RS) compared to BYAT526 (*YALI* Lys-RS / *SACE* Lys-RS) . Red dots: genes involved in lysinyl biosynthetic pathway (right box, fold excess as above). Black dots: genes involved in pathway regulation (bottom box). Fold excess of RNA levels computed from M NORM.  Note the very severe phenotypic defect of BYAT581-0 (Supplementary Fig. 10). Biosynthetic pathways were taken from SGD YeastCyc Biochemical pathways http://pathway.yeastgenome.org.

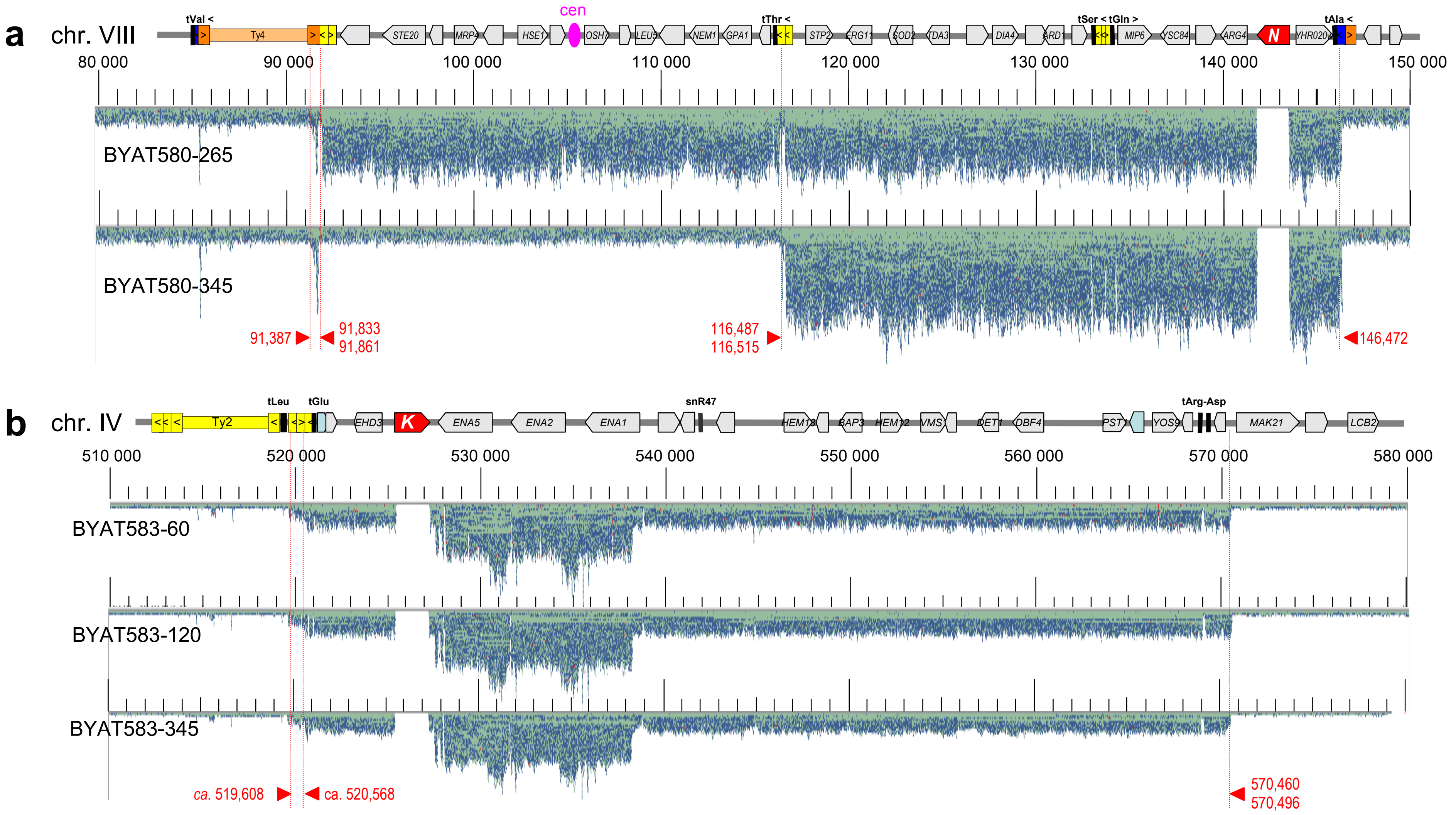


**Supplementary Figure 4: *Tablet*[4] images of Illumina HiSeq deep sequencing of evolved strains mapped to the reference S288c genome sequence.**

**a.** Segment of chromosome VIII (from coordinate 80,000 to 150,000). Major genetic elements annotated along this segment are cartooned on top of the figure (legend as Fig. 4). BYAT580-265 and -345 were sequenced at average depths of 263 X and 322 X, respectively. Note the similar amplification (12 copies in excess of diploid number) for the very short (91,387-91,861) and the large (116,487-146,472) segments in BYAT580-345 and the perfect coincidence with limits of the amplified segment in BYAT580-265. For coordinates, see Supplementary Fig. 9.

**b.** Segment of chromosome IV (from coordinate 510,000 to 580,000). Major genetic elements annotated along this segment are cartooned on top of the figure. Same legend. BYAT583-60, -120 and -345 were sequenced at average depths of 384 X, 440 X and 296 X, respectively. The 50kb segment (519,608-570,496) is amplified in 20-22 copies in excess to diploid number. Note the half-level amplification at its left border (*ca.* 519,608 - *ca.* 520,568), and the excess coverage of the segment corresponding to the tandem repeat of *ENA* genes (9 copies in our strains, compared to 3 copies in the reference sequence). The left border is a quasi-palindromic junction between *YDRCdelta6a* (520,173 - 520, 475) and *YDRWdelta7* (520,477 - 520,805). The right border is a quasi-palindromic junction between two non-identical sequences of the bidirectional promoter segment separating *YDR059c* from *YDR060w* (see Fig. 4).

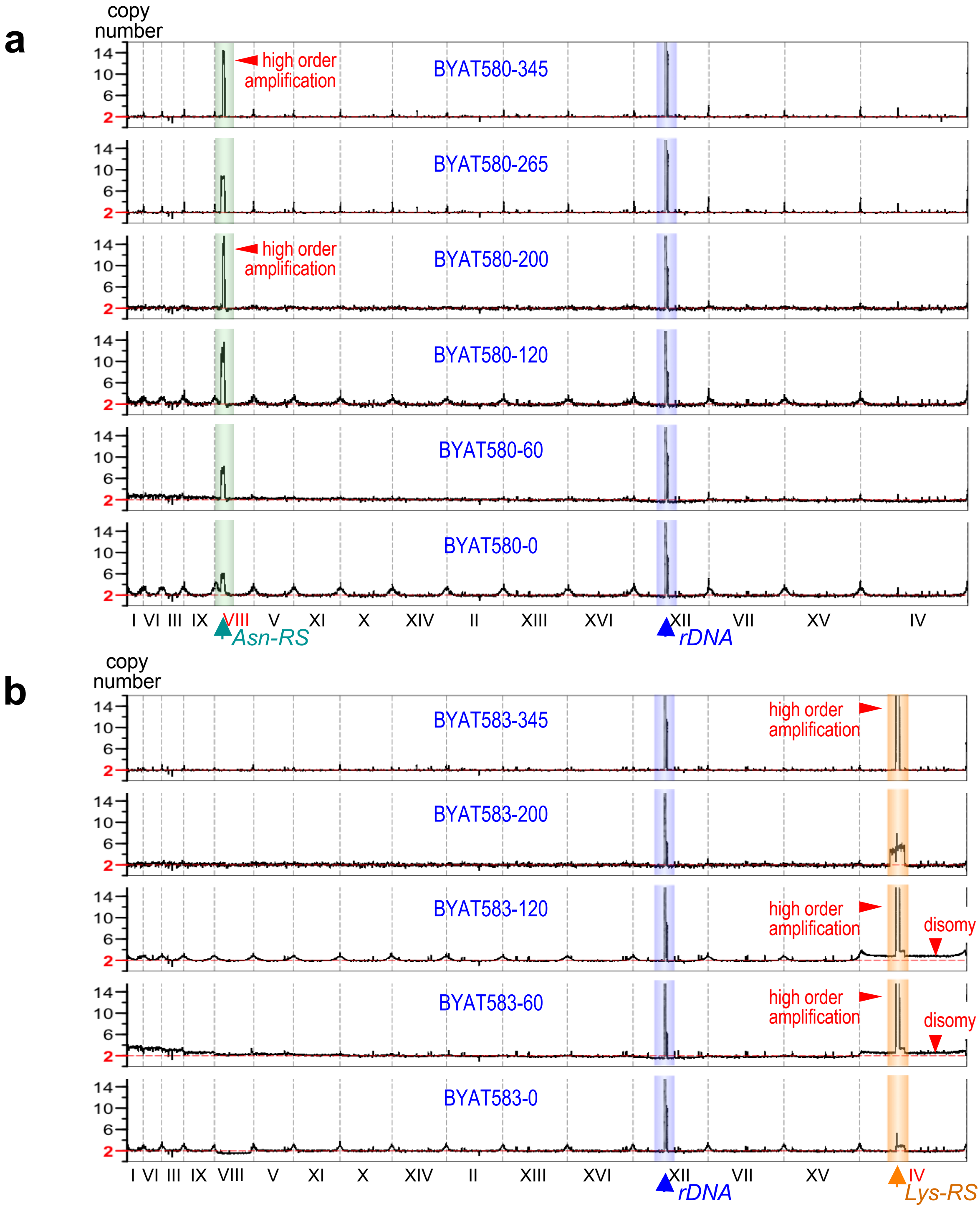


**Supplementary Figure 5: Copy number variation along chromosomes of parental and evolved strains as deduced from sequencing coverages.**
For each sequenced strain, the copy number of each locus (ordinate) was plotted along the 16 chromosomes of the S288c reference sequence ranked by increasing sizes (abscissa, roman numerals). Copy numbers were computed from local sequence coverage relative to the median coverage for that strain, and normalized to 2 (diploids). Curve smoothing (5,000 bp sliding-windows). Amplification of the rDNA locus (blue background) was artificially cut for drawing clarity. *CUP1* and *URA3* loci were ignored (presence of tandem repeats and multicopy plasmids).
**a.** BYAT580 evolutionary experiment. Position of the *YALI* Asn-RS gene is indicated (chromosome VIII) and amplification of the corresponding region is highlighted by turquoise background.
**b.** BYAT583 evolutionary experiment. Position of the *YALI* Lys-RS gene is indicated (chromosome IV) and amplification of the corresponding region is highlighted by orange background. Note the three copies of chromosome IV in strains BYAT583-60 and -120 and the absence of other visible copy number variation. Excess coverage of chromosomes I, VI and III in BYAT583-60 is an artifact of DNA purification.

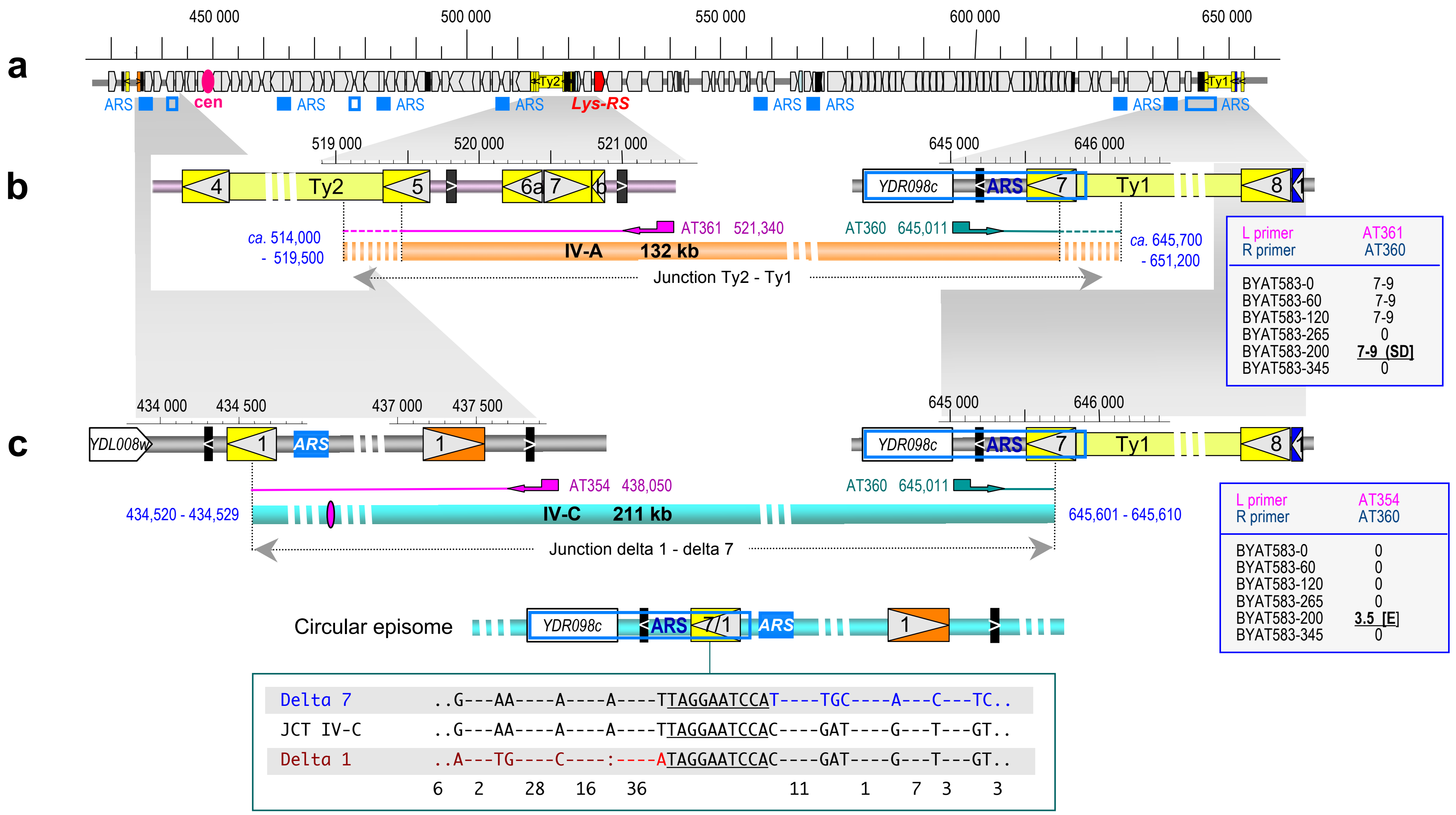


**Supplementary Figure 6: Analysis of amplicon IV-A and IV-C junctions in the BYAT583 evolutionary experiment.**
**a.** Segment of chromosome IV in which both amplificons were found (legend as in Fig. 4).
**b.** Mappig of amplicon IV-A junction by PCR amplification. Orange bar: amplicon unit. PCR primers (magenta and turquoise arrows) are indicated by numbers (ATnnn, Supplementary Table 3) with their 5' end coordinates. Thin lines indicate sequenced PCR product (dotted lines, unsequenced parts). Right box: size of PCR products in kb (bold underlined: product was sequenced). 0: no amplfication. The segmental duplication (SD) of amplicon IV-A occurred between *YDRCTy2-1* (513,692 - 519,647) and *YDRCTy1-1* (645,502 - 651,419), two co-oriented elements along chromosome IV that exhibit 22% sequence divergence over their entire lengths.
**c.** Mapping of amplicon IV-C junction by PCR amplification. Turquoise bar: amplicon unit. Same legend as b. Bottom: junction in the circular episome. Box: alignment of junction sequence (black letters) with *YDLCdelta1* (coordinates 434,423 - 434,739) and *YDRCdelta7* (645,502 - 645,835), two co-oriented elements on chromosome IV showing 21 % sequence divergence over their entire lengths. Diverging sites are shown with their distance (bottom) in nucleotides. (:) indel. The junction occurred within the 10-nucleotide long segment (underlined sequence).

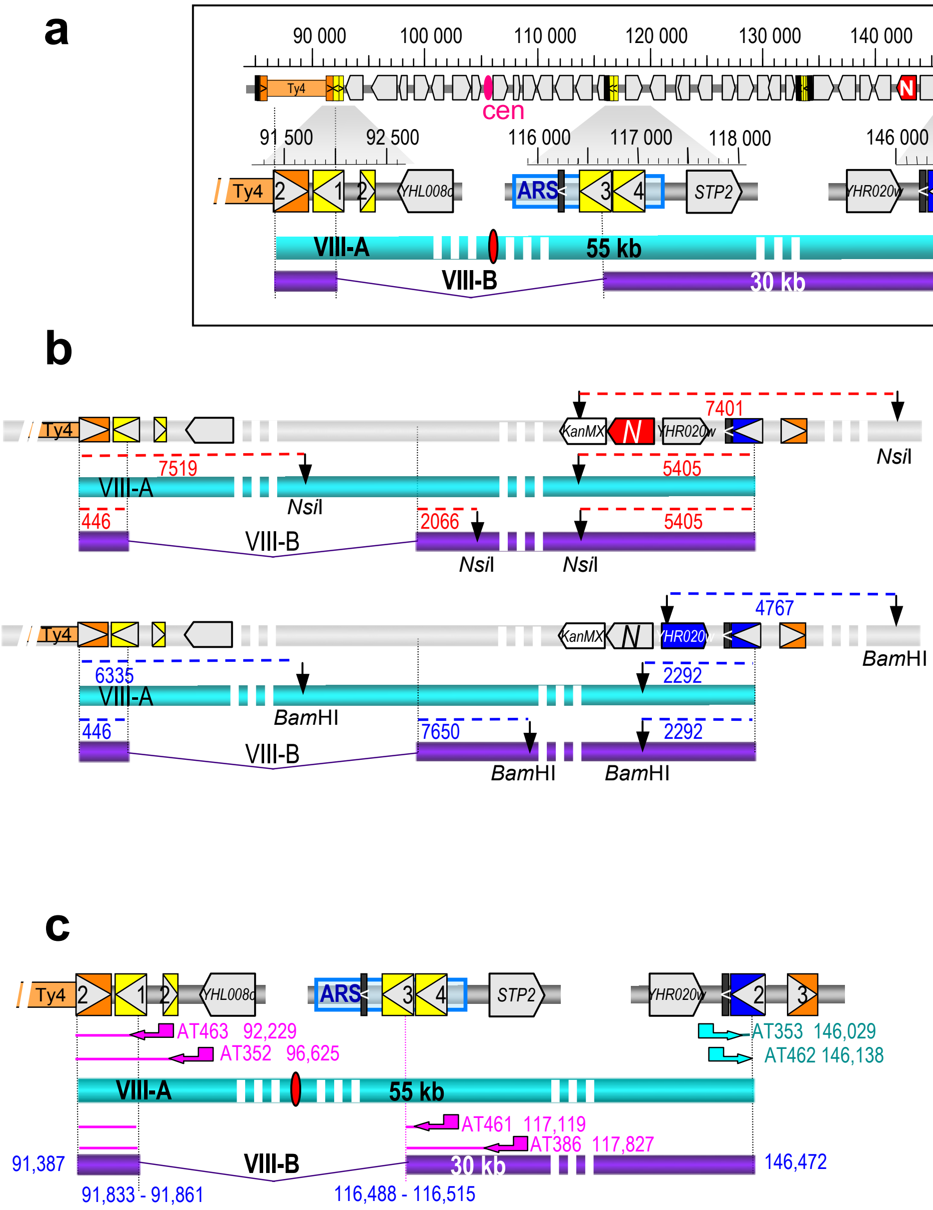


| Nsi I digest | Asn-RS probe | |
|---|---|---|
| BYAT580-0 | 7.4 | 12.9 |
| BYAT580-60 | 7.4 | 12.9 |
| BYAT580-120 | 7.4 | 12.9 |
| BYAT580-265 | 7.4 | 12.9 |
| BYAT580-200 | 7.4 | 7.9 |
| BYAT580-345 | 7.4 | 7.9 |

| *Bam*HI digest | *YHR020w* probe | |
|---|---|---|
| BYAT580-0 | 4.8 | 8.8 |
| BYAT580-60 | 4.8 | 8.8 |
| BYAT580-120 | 4.8 | 8.8 |
| BYAT580-200 | 4.8 | 10.4 |

| L primer / R primer | AT352 / AT353 | AT463 / AT462 | AT386 / AT353 | AT461 / AT462 |
|---|---|---|---|---|
| BYAT580-0 | **1.7** | **1.3** | 0 | |
| BYAT580-60 | 1.7 | **1.3** | 0 | 0 |
| BYAT580-120 | 1.7 | **1.3** | 0 | 0 |
| BYAT580-265 | 1.7 | **1.3** | 0 | 0 |
| BYAT580-200 | 0 | 0 | **2.2** | **1.4** |
| BYAT580-345 | | | 2.2 | **1.4** |

**Supplementary Figure 7: Analysis of amplicon junctions in the BYAT580 evolutionary experiment.**
**a.** Segment of chromosome VIII in which amplicons were found with details of genetic elements at amplicon border regions (legend as in Fig. 4). Colored bars: amplicon units.
**b.** Mapping of amplicon junctions by genomic digests and *Southern* blot hybridizations. Grey bar: normal chromosome with summary of relevant elements, Turquoise and purple bars: amplicons VIII-A and VIII-B, respectively. Sequence-deduced restriction fragment sizes (horizontal broken lines between vertical arrows) are indicated in nucleotides. Right boxes: size of hybridizing bands on gels in kb. Left columns: wild-type bands, right columns: amplicon junctions. Note the absence of 12.9 kb and 8.8 kb junction fragments in BYAT580-200, excluding a possible location of the tandem array at coordinate 91,387 on the left arm.
**c.** Mapping of amplicon junctions by PCR amplifications. PCR primers (magenta and turquoise arrows) are indicated by numbers (ATnnn, Supplementary Table 3 ) with their 5' end coordinates. Thin lines indicate sequenced products. Right box: size of PCR products in kb (bold underlined: product was sequenced). 0: no amplification. Void*:* not done.

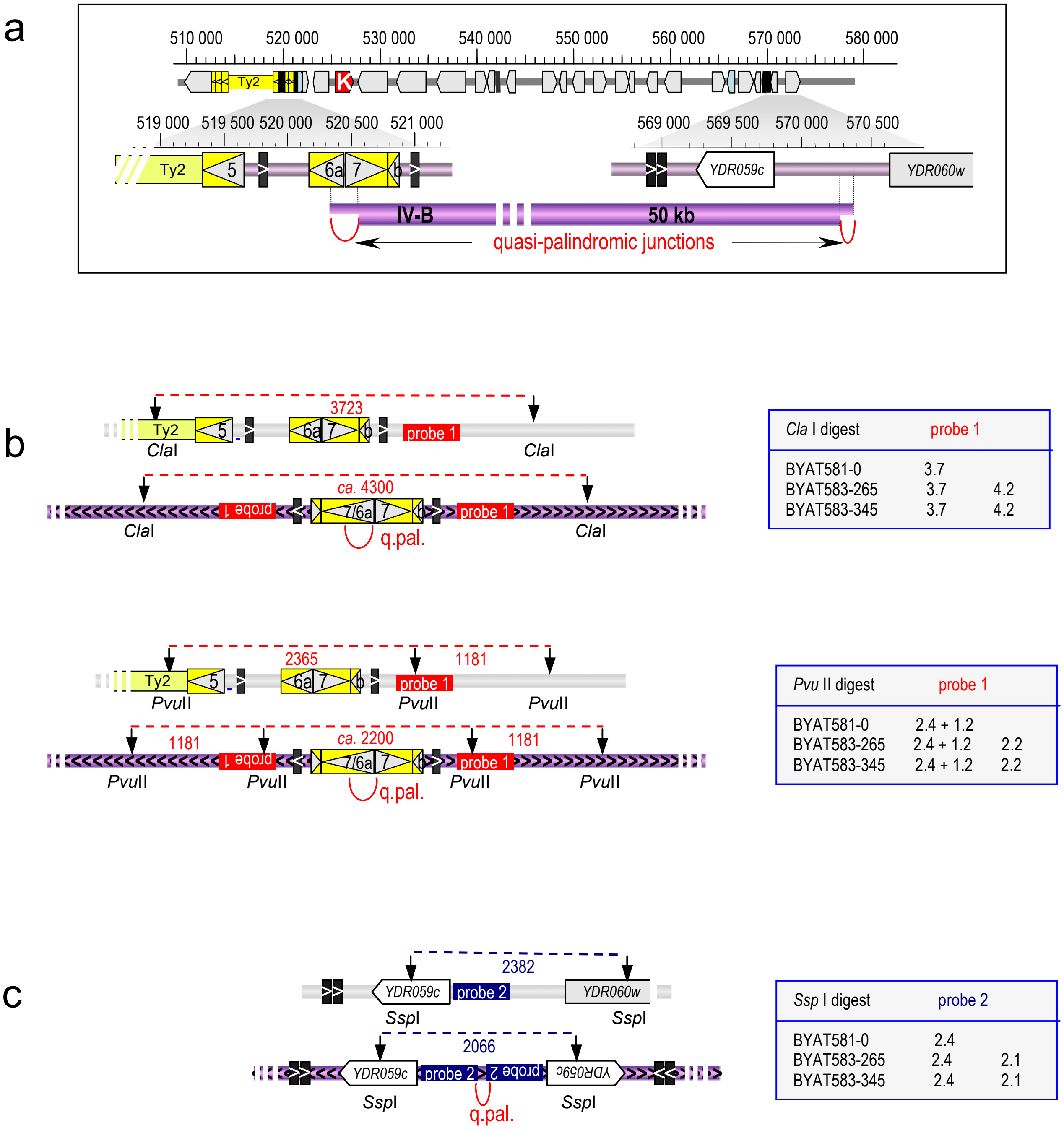


**Supplementary Figure 8: Analysis of amplicon IV-B junctions in the BYAT583 evolutionary experiment.**
**a.** Segment of chromosome IV in which amplicon IV-B was found with details of genetic elements at amplicon border regions (legend as in Fig. 4). Purple bar: amplicon unit.
**b.** Mapping of left junction by genomic digests and *Southern* blot hybridations. Grey bar: normal chromosome. Purple bar with arrows: quasi-palindromic left junction. Sequence-deduced restriction fragment sizes (horizontal broken lines between vertical arrows) are indicated in nucleotides. Right boxes: size of hybridizing bands on gels in kb. Left columns: wild-type bands, right columns: amplicon junctions. *Pvu*II fragment size agrees with a quasi-palindromic junction within the 42 nt long common sequence between *YDRCdelta6a* (520,173 - 520,475) and *YDRWdelta7* (520,477 - 520,805), two inverted adjacent elements on chromosome IV that exhibit 23 % sequence divergence over their entire length.
**c.** Mapping of right junction, same legend. Fragment size agrees with the quasi-palindromic junction identified from genome sequence assembly of BYAT583-345 (Fig.4).

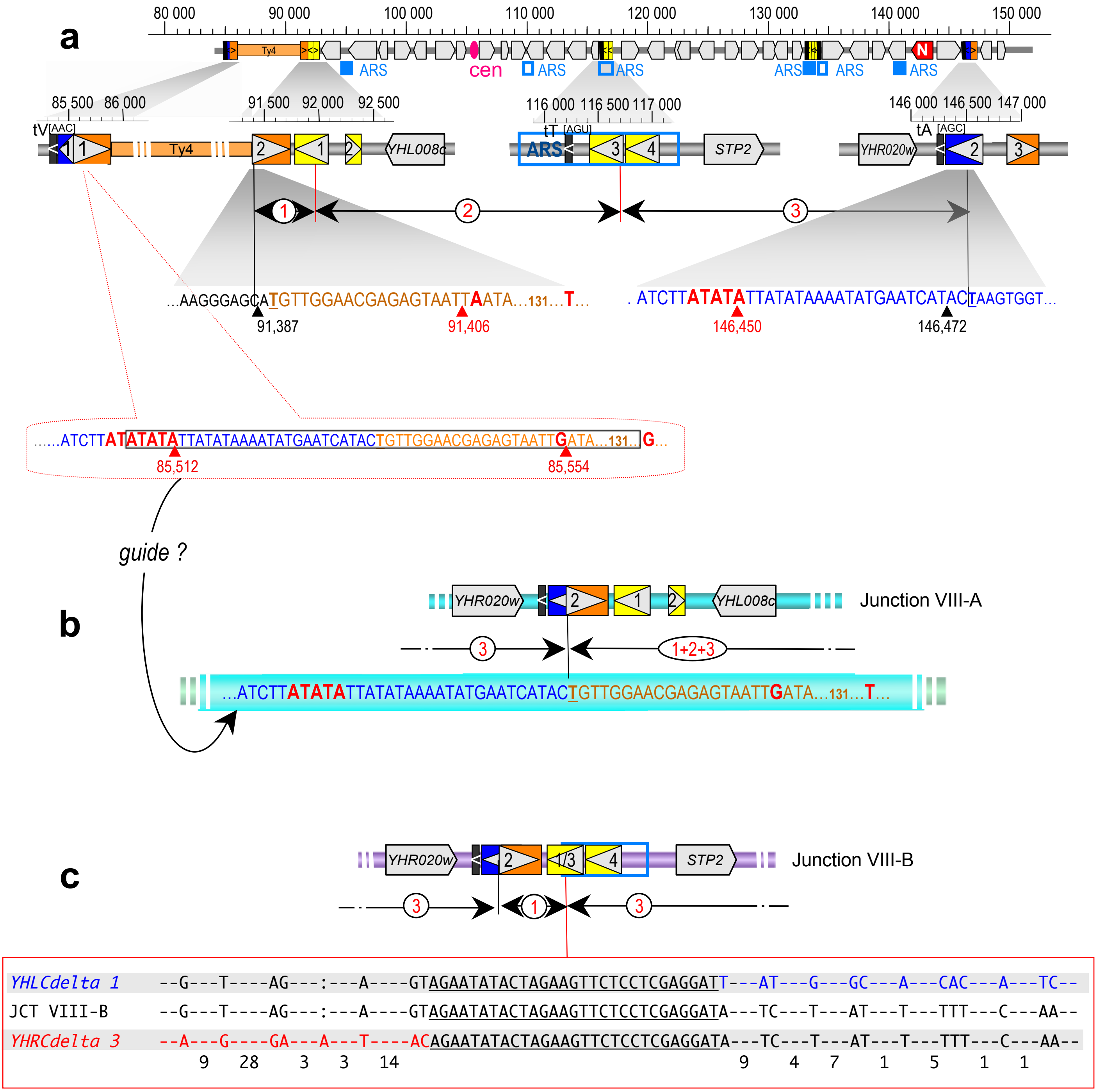


**Supplementary Figure 9. Formation of amplicon VIII-A and VIII-B junctions in the BYAT580 evolutionary experiment.**
**a.** Segment of chromosome VIII in which amplicons were found. Legend as in Fig. 4. Junction regions are zoomed. Breakpoints define three sections (double arrows with circled numbers). Thin black vertical lines: breakpoints of amplicons VIII-A and VIII-B, red vertical lines: breakpoints of amplicons VIII-B. Sequences correspond to parental strain (determined from BYAT580-0, identical to S288c reference sequence). Blue letters: *sigma* elements; orange letters: *tau* elements; bold red letters: sequence divergence with coordinates.
**b.** Amplicon VIII-A junction in circular episomes of evolved mutants BYAT580-60, -120 and -265. Sequence color code as above). Note the identity with the possible « guide » sequence upstream of *YHLWTy4-1* shown in a. Boxed letters: maximal possible extension as determined from sequence divergences (bold red letters).
**c.** Internal junction between sections 1 and 3 in amplicon VIII-B within *macrotene* chromosome of evolved mutants BYAY580-200 and -345. Box: alignment of junction sequence (black letters) with *YHLCdelta1* and *YHRCdelta3*, two co-oriented elements on chromosome VIII showing 21 % sequence divergence over their entire lengths. Diverging sites are shown with their distance (bottom) in nucleotides. (:) indel. The junction occurred within the 29-nucleotide long segment (underlined sequence).

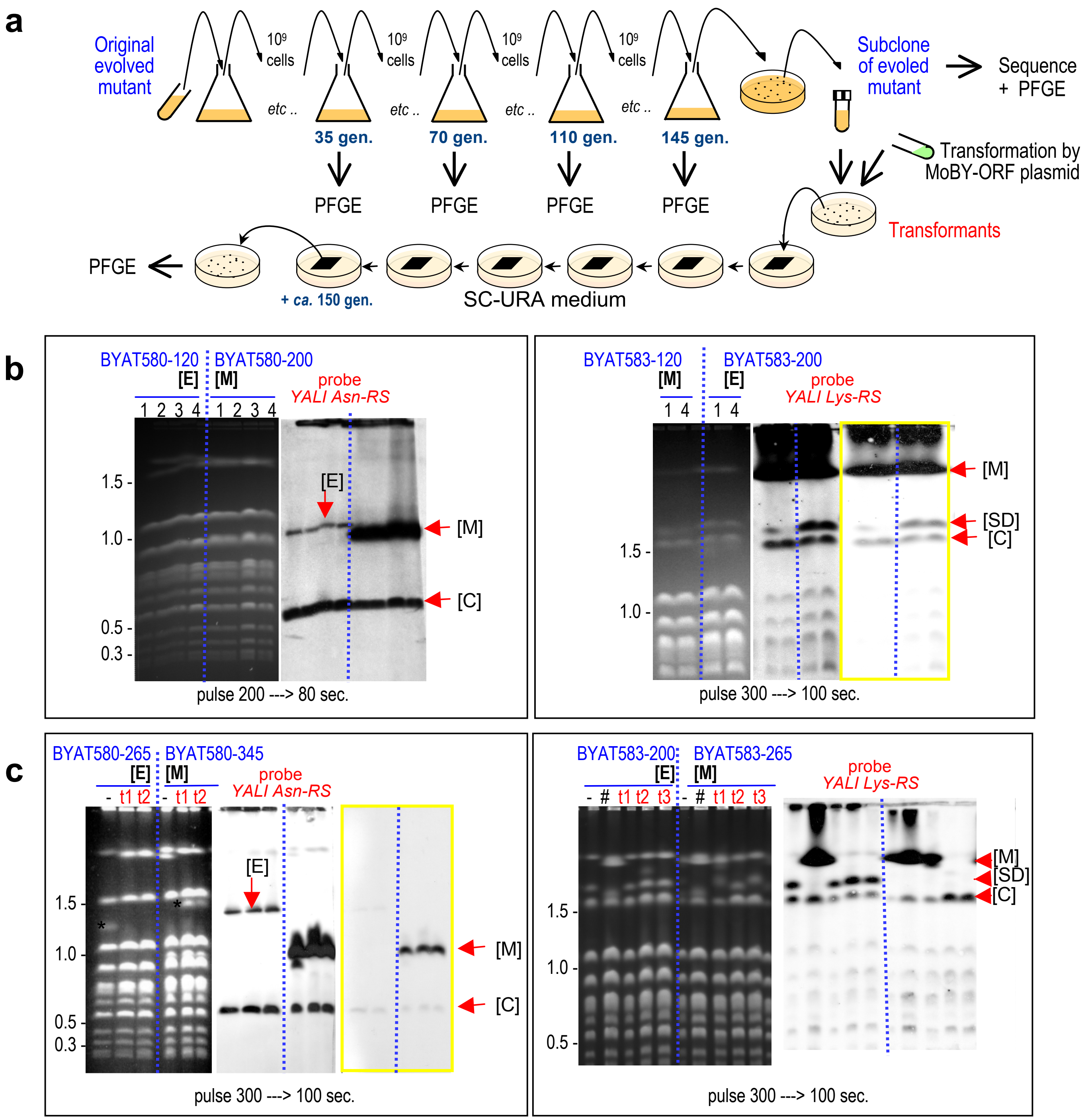


**Supplementary Figure 10: Stability of amplified structures in evolved mutants.**
**a**. Experimental set up. Original evolved mutants (BYAT580-120, -200, and BYAT583-120, -200) were grown for total of 145 generations on YPD medium by the serial transfer method with regular monitoring of populations by PFGE, after which subclones were isolated at random and sequenced. Interesting subclones (BYAT580-265, 345, and BYAT583-201, -265) were transformed by plasmids of the MoBY-ORF collection [5] bearing either the Asn-RS or the Lys-RS gene of *S. cerevisiae*, as appropriate. Transformants were propagated on SC-URA plates (to maintain plasmids) without subcloning for *ca.* additional 150 generations, after which random subclones (t1, t2, t3) were tested by PFGE.
**b.** Stability of evolved mutants analyzed by PFGE (1, 2, 3, and 4 correspond, respectively, to populations after 35, 70, 110 and 145 generations). All PFGE were performed as described in Methods. Left panels: ethidium bromide fluorescence. Size scale in Mb, calibrated from migration of natural *S. cerevisiae* chromosomes. Right panels: hybridization with probes. Yellow borders: under-exposition to facilitate visualization. [C] normal chromosome, [M] *macrotene* chromosome, [E] episome, [SD] segmental duplication.
**c**. Stability of evolved mutant subclones before and after tranformation by MoBY-ORF plasmids. -: non-transformed evolved mutant, # *id.* after *ca.* 150 additional generations on YPD medium. t1, t2, t3 subclones of transformants after *ca.* 150 generations on SC-URA medium. Yellow borders: under-exposition to facilitate visualization. * altered chromosome XII migration (rDNA repeats, hybridization not shown).

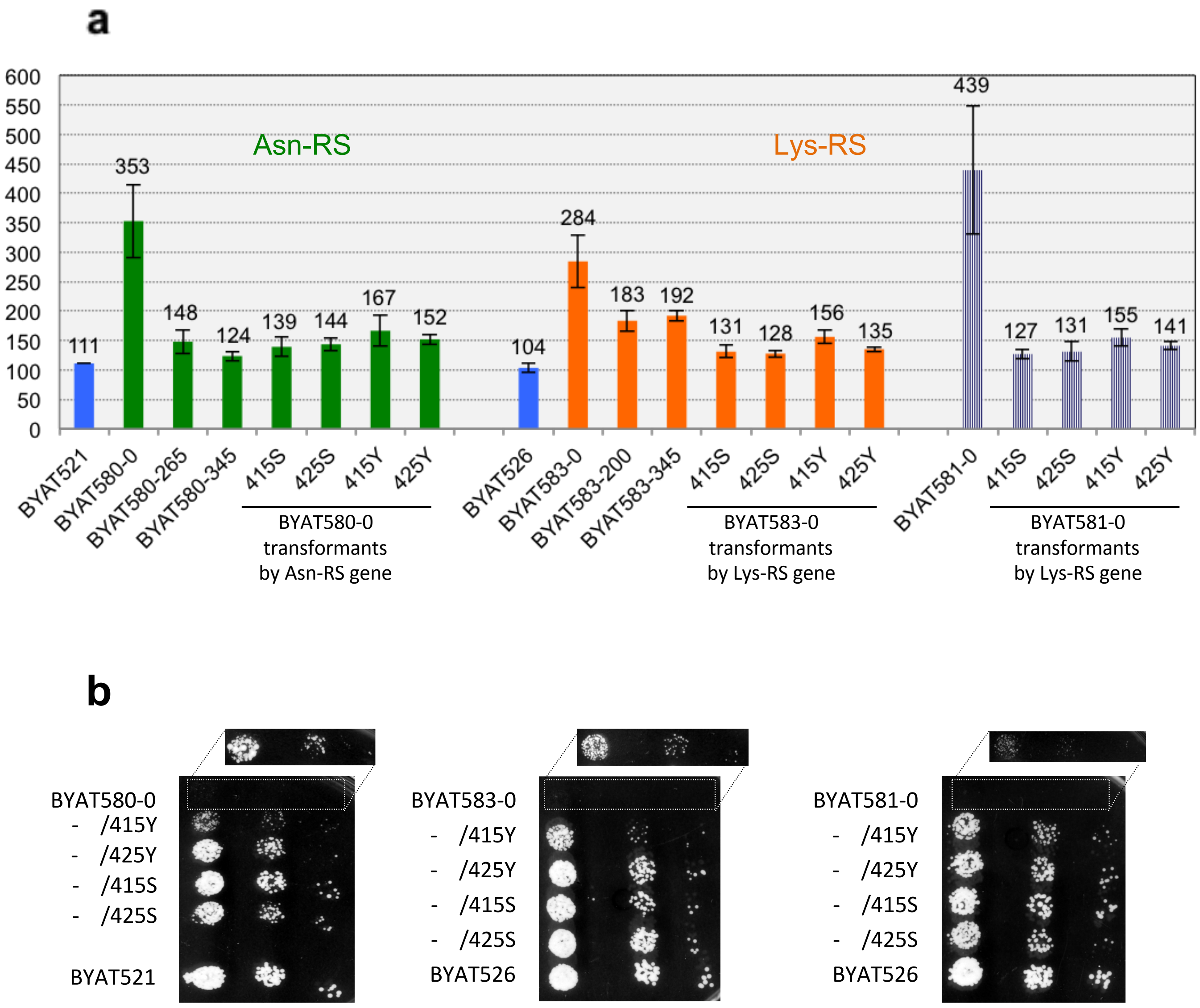


**Supplementary Figure 11. Generation time of parental and evolved strains, and phenotopic restoration by *S. cerevisiae* and *Y. lipolytica* RS genes.**

**a.** Average generation times (expressed in minutes, ordinate) were calculated from OD values comprised between 0.2 to 0.4 according to Methods. For each strain (parental, evolved mutant or transformant), four to twelve subclones (two subclones for controls BYAT521 and BYAT526) were inoculated into 150 µl of fresh YPD medium in 96-well microtiter plates for automated OD measurements. Original data are available in Figshare http://figshare.com/s/6b33ab5a7af011e4bede06ec4bbcf141. Error bars represent 99% confidence limits (± 2.6 σ / √(n-1), where n is the number of subclones studied for each strain). Transformants (see Methods) were constructed using *E.coli-S. cerevisiae* shuttle vectors pRS415 (centromeric) or pRS425 (multicopy) [3], bearing Asn-RS or Lys-RS genes from *Y. lipolytica* (Y) or *S. cerevisiae* (S).

**b.** Drop tests of parental strain subclones and their transformants compared to control strains (BYAT521 and BYA526). YPD medium (note possible plamid loss). Pictures taken after 30 hours of incubation at 30°C, and again at 90 hours of incubation for non-transformed strains top).

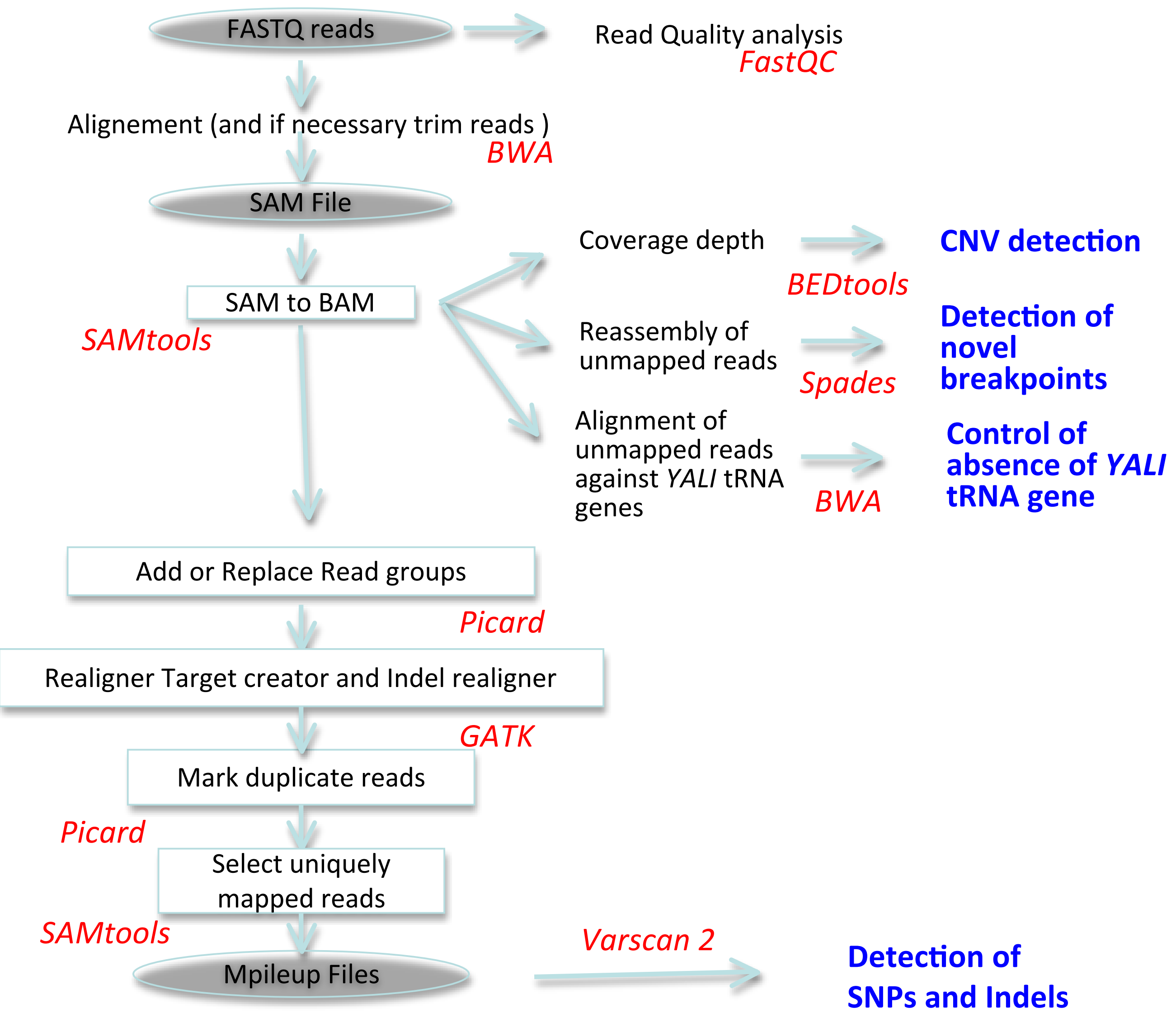


**Supplementary Figure 12: General pipeline for deep sequencing analysis.**
Files are indicated by shaded ovals and softwares by red fonts. See Methods for details.

## Supplementary Table 1: List of initial strains

| Strain | Ploidy | Nuclear genotype (+ transforming plasmid, see Supplementary Information) | Origin |
|---|---|---|---|
| BYAT290 | 2n | *MATa/MATα ura3Δ0 /ura3Δ0 leu2Δ0/leu2Δ0 his3Δ1/his3Δ1 lys2Δ0/+ met15Δ0/+ can1::HygR/+* | Cross YBaG305 x BY4742 [(1)] |
| **Construction of Asn-RS strains** | | | |
| BYAT521 | 2n | Same as BYAT290, except that one allele of *YHR019c* was replaced by the *YALI0E05005g-KanMX* construct (see Supplementary Fig. 1) | Integrative transformant of BYAT290 |
| BYAT561 | n | *MATα ura3Δ0 leu2Δ0 his3Δ1 lys2Δ0* ***YHR019c::YALI0E05005g-KanMX*** (pAT260) | Meiosis of BYAT521 [pAT260] |
| BYAT562 | n | *MATa ura3Δ0 leu2Δ0 his3Δ1 met15Δ0* ***YHR019c::YALI0E05005g-KanMX*** | Meiosis of BYAT521 [pAT260] |
| **BYAT580** | 2n | *MATa/MATα ura3Δ0 /ura3Δ0 leu2Δ0/leu2Δ0 his3Δ1/his3Δ1 lys2Δ0/+ met15Δ0/+* ***YHR019c::YALI0E05005g-KanMX / YHR019c::YALI0E05005g-KanMX*** (pAT260) | Mating of BYAT561 and BYAT562 |
| **Construction of Lys-RS strains** | | | |
| BYAT526 | 2n | Same as BYAT290, except that one allele of *YDR037w* was replaced by the *YALI0F16291g -KanMX* construct (see Supplementary Fig. 1) | Integrative transformant of BYAT290 |
| BYAT565 | n | *MATα ura3Δ0 leu2Δ0 his3Δ1 lys2Δ0* ***YDR037w::YALI0F16291g-KanMX*** (pAT270) | Meiosis of BYAT526 [pAT270] |
| BYAT566 | n | *MATa ura3Δ0 leu2Δ0 his3Δ1 met15Δ0* ***YDR037w::YALI0F16291g- KanMX*** (pAT270) | Meiosis of BYAT526 [pAT270] |
| **BYAT581** | 2n | *MATa/MATα ura3Δ0 /ura3Δ0 leu2Δ0/leu2Δ0 his3Δ1/his3Δ1 lys2Δ0/+ met15Δ0/+* ***YDR037w::YALI0F16291g-KanMX / YDR037w::YALI0F16291g-KanMX*** | Mating of BYAT565 and BYAT566 |
| BYAT571 | n | *MATa ura3Δ0 leu2Δ0 his3Δ1 met15Δ0* ***YDR037w::YALI0F16291g-KanMX*** | Meiosis of BYAT526 [pAT272] |
| BYAT572 | n | *MATα ura3Δ0 leu2Δ0 his3Δ1 lys2Δ0* ***YDR037w::YALI0F16291g-KanMX*** | Meiosis of BYAT526 [pAT274] |
| **BYAT583** | 2n | *MATa/MATα ura3Δ0 /ura3Δ0 leu2Δ0/leu2Δ0 his3Δ1/his3Δ1 lys2Δ0/+ met15Δ0/+* ***YDR037w::YALI0F16291g-KanMX / YDR037w::YALI0F16291g-KanMX*** | Mating of BYAT571 and BYAT572 |

Strains BYAT561 and BYAT562 were derived from a transformant of the diploid BYAT521 by the multicopy plasmid pAT260. BYAT561 and BYAT580 kept the plasmid while BYAT562 lost it during meiosis. Strains BYAT565 and BYAT566 were derived from a transformant of the diploid BYAT526 by the multicopy plasmid pAT270 and originally kept it. This plasmid, however, was lost in the subclones used to construct BYAT581. Strains BYAT571 and BYAT572 were derived from transformants of the diploid BYAT526 by the multicopy plasmids pAT272 and pAT274, but lost them during meiosis. All plasmids are derivative of pRS426, a 2μ-plasmid based *E.coli-S.cerevisiae* shuttle vector containing the *URA3* marker [3]. Each plasmid was constructed by insertion at the *Bam*H1 site of a Phusion-PCR product of a short *Y. lipolytica* genomic segment bearing an Asn$^{[GUU]}$ tDNA (pAT260), a Lys$^{[UUU]}$ tDNA (pAT272 and pAT274) or a Lys$^{[UUU]}$-Glu [CUC] tDNA transcriptional fusion (pAT270). Diploid strains used for this work are bold-type underlined.

(1) Strain YBaG305 is an integrative transformant of strain BY4741 in which the *CAN1* gene (*YEL063c*) was replaced by a hygromycine-resistant cassette (unpublished). Strains BY4741 and BY4742 are S288c derivatives [5].

**Supplementary Table 2: Non-synonymous nucleotide substitutions in sequenced strains**

| # | evolved mutant(s) | chr. nb | seq. coord. | ref | mut | systematic number | gene name | codon ref. | codon mut. | aa. change | brief functional description |
|---|---|---|---|---|---|---|---|---|---|---|---|
| N01 | BYAT580-0, -60, -120, -200, -265, -345 | 1 | 41 420 | G | A | *YAL056W* | *GPB2* | GAG | AAG | E > K | Multistep regulator of cAMP-PKA signaling, ohnolog of *GPB1* |
| N02 | BYAT580-0, -60, -120, -200, -265, -345 | 3 | 135 629 | G | C | *YCR011C* | *ADP1* | CCC | GCC | P > A | Putative ATP-dependent permease of the ABC transporter family |
| N03 | BYAT580-0, -60, -120, -200, -265, -345 | 3 | 268 233 | C | A | *YCR089W* | *FIG2* | CCA | CAA | P > Q | Cell wall adhesin, ohnolog of *AGA1* |
| N04 | BYAT580-0, -60, -120, -200, -265, -345 | 4 | 115 664 | A | C | *YDL193W* | *NUS1* | GAU | GCU | D > A | Putative prenyltransferase; required for cell viability |
| N05 | BYAT580-0, -60, -120, -200, -265, -345 | 7 | 448 977 | A | G | *YGL025C* | *PGD1* | CUA | CCA | L > P | Subunit of the RNA polymerase II mediator complex; forms the RNA polymerase II holoenzyme. |
| N06 | BYAT580-0, -60, -120, -200, -265, -345 | 7 | 583 596 | T | C | *YGR044C* | *RME1* | AUA | GUA | I > V | Zinc finger protein involved in control of meiosis; relocalizes from nucleus to cytoplasm upon DNA replication stress |
| N10 | BYAT580-60 | 13 | 577 972 | A | G | *YMR161W* | *HLJ1* | AGG | GGG | R > G | Co-chaperone for Hsp40p; anchored in the ER membrane |
| N11 | BYAT580-60 | 15 | 877 471 | C | A | *YOR298C-A* | *MBF1* | GUC | UUC | V > F | stress |
| N12 | BYAT580-200, -345 | 9 | 352 510 | G | T | *YIL002C* | *INP51* | CAA | AAA | Q > K | Phosphatidylinositol 4,5-bisphosphate 5-phosphatase |
| N13 | BYAT580-200, -345 | 13 | 554 216 | A | G | *YMR144W* | | AAC | GAC | N > D | Putative protein of unknown function; localized to the nucleus |
| N14 | BYAT580-265 | 14 | 400 509 | C | T | *YNL121C* | *TOM70* | GCC | ACC | A > T | Component of the TOM complex, ohnolg of *TOM71* |
| N15 | BYAT580-345 | 2 | 569 602 | G | T | *YBR165W* | *UBS1* | AGG | AGU | R > S | Ubiquitin-conjugating enzyme suppressor, positive regulator of Cdc34p activity |
| K01 | BYAT583-0, -60, -120, -200, -345 | 4 | 594 391 | T | G | *YDR074W* | *TPS2* | AAU | AAG | N > K | Phosphatase subunit of the trehalose-6-P synthase/phosphatase complex, induced by DNA replication stress conditions |
| K02 | BYAT583-0, -60, -120, -200, -345 | 12 | 403 424 | T | A | *YLR130C* | *ZRT2* | AAU | UAU | N > Y | Low-affinity zinc transporter of the plasma membrane |
| K03 | BYAT583-0, -60, -120, -200, -345 | 12 | 421 168 | T | A | *YLR138W* | *NHA1* | UAC | AAC | Y > N | Na+/H+ antiporter |
| K04 | BYAT583-0, -60, -120, -200, -345 | 14 | 189 586 | C | T | *YNL243W* | *SLA2* | UCC | UUC | S > F | Adaptor protein that links actin to clathrin |
| K05 | BYAT583-0, -60, -120, -200, -345 | 14 | 660 537 | G | C | *YNR016C* | *ACC1* | AAC | AAG | N > K | Acetyl-CoA carboxylase, ohnolog of *HFA1* |
| K06 | BYAT583-0, -60, -120, -200, -345 | 15 | 648 906 | C | G | *YOR167C* | *RPS28A* | GAC | CAC | D > H | Protein component of the small (40S) ribosomal subunit, ohnolog of *RPS28B* |
| K10 | BYAT583-0 | 2 | 234 714 | C | A | *YBL004W* | *UTP20* | GCG | GAG | A > E | Component of the small-subunit (SSU) processome involved in the biogenesis of the 18S rRNA |
| K11 | BYAT583-60 | 5 | 519 339 | A | C | *YER167W* | *BCK2* | ACG | CCG | T > P | Ser/Thr-rich protin involved in *PKC1* signalling pathway |
| K12 | BYAT583-60 | 8 | 98 628 | A | C | *YHL006C* | *SHU1* | GUG | GGG | V > G | Component of the Shu complex, which pomotes error-free DNA |
| K13 | BYAT583-120 | 9 | 105 430 | T | A | *YIL130W* | *ASG1* | AAU | AAA | N > K | Regulator involved in stress response, inc cluster protein |
| K14 | BYAT583-120 | 14 | 312 337 | A | T | *YNL172W* | *APC1* | GAC | GUC | D > V | Largest subunit of anaphase-promoting complex, responds to DNA replicatioon stress |
| K15 | BYAT583-200 | 4 | 626 778 | C | G | *YDR091C* | *RLI1* | AAG | AAC | K > N | RNase L Inhibitor |
| K16 | BYAT583-200 | 15 | 541 975 | C | A | *YOR116C* | *RPO31* | AAG | AAU | K > N | RNA polymerase III largest subunit C160 of core enzyme |
| K17 | BYAT583-200 | LYS | 1 764 | G | A | *YALI0F16291g* | | GAA | AAA | E > K | Y. lipolytica Lysinyl tRNA synthetase |
| K18 | BYAT583-345 | 4 | 1 287 320 | G | T | *YDR407C* | *TRS120* | AAC | AAA | N > K | Component of transport protein particle (TRAPP) complex II |
| K19 | BYAT583-345 | 11 | 620 894 | A | C | *YKR095W* | *MLP1* | CAA | CCA | Q > P | Myosin-like protein associated with the nuclear envelope, ohnolog of *MLP2* |
| K20 | BYAT583-345 | 16 | 323 270 | G | T | *YPL120W* | *VPS30* | GAA | UAA | E > stop | Subunit of phosphatidylinositol (PtdIns) 3-kinase complexes I and II |

Non-synonymous nucleotide substitutions in annotated CDS were identified from complete genome sequences as described in Methods. Each mutation was arbitrarily numbered (column 1) and the list of strains sharing the mutation is in column 2. All mutations are in heterozygous form with the wild-type allele, except for K04 that must have arose during BYAT526 strain construction (see Supplementary Table 1). Mutation K17 affected 3 out of 5 copies of the *Y. lipolytica* Lys-RS gene in BYAT583-200 according to its frequency among sequence reads, suggesting that the mutational event occured on the episome. Its coordinate refers to *YALI0F16291g*. Functional descriptions taken from http:// www.yeastgenome.org.

**Supplementary Table 3: Sequence of primers used in this work.**

| primer n° | DNA sequence (5' - 3') |
|---|---|
| AT200 | GGGTCCTCACGCAAGTAGCAAGGTAATCTATATCCTCATTTTTTTCAAACATGAGTCAAAGTTCGTCTGA |
| AT201 | GATTCAGATCCACTAGTGGCTTAAGGCTTGCATCTGCCAG |
| AT202 | CTGGCAGATGCAAGCCTTAAGCCACTAGTGGATCTGAATC |
| AT203 | TAAATATTTGTATATATGTGAGCGCTCACGTACTCTTCAGTAACTAAAGAGCTGAAGCTTCGTACGCTGC |
| AT214 | TATTGCTCTGTCCTTCCTCCGAGAAATCCGTAATAAACTTCAATAGCATAATGTCTGATATTAAGGAAACTACCG |
| AT215 | GATTCAGATCCACTAGTGGCCTACTCCTGCTGCTCCTTCTTATCG |
| AT216 | CGATAAGAAGGAGCAGCAGGAGTAGGCCACTAGTGGATCTGAATC |
| AT217 | CTTAGAATAATAATACTTACATTATACAATATGTTCTAATATCTTTTAGCGCTGAAGCTTCGTACGCTGC |
| AT352 | TGACCAGAAGAGTTAAGATG |
| AT353 | TACTGTATGTTCGGTCGTTC |
| AT354 | GTCCAGATGATACACCTTGT |
| AT360 | GATGGAACCTGAAAAGAACAC |
| AT361 | GCATAATGCATATTTTGATGATGC |
| AT386 | ATAGTGATAAGATAGGCAT |
| AT461 | TATCACGACATGTGCGCTT |
| AT462 | TCAAATAATGTTTCCGCTAT |
| AT463 | GGCATGTGTTCGGAACATA |

Sequences of synthetic oligonucleotides used as primers for PCR amplifications reported in Supplementary Figs. 1, 6 and 7.

## Supplementary Note 1: Choice of tRNA synthetase genes and donor yeast species

The normal set of cytosolic and/or mitochondrial tRNA synthetases (RS) of *S. cerevisiae* is encoded by a total of 36 genes [6], among which only 12 are dedicated to purely cytosolic enzymes (distinct from their mitochondrial counterparts) made of monomeric or homopolymeric subunits, a critical criterion to facilitate interspecific gene replacement. Considering the number of distinct isoacceptor tRNA species charged by each RS (from 1 to 3) [7], we narrowed down our primary selection to three class I enzymes (for Glu, Trp and Tyr, respectively) and three class II enzymes (for Asn, Asp, and Lys, respectively). Each one is encoded by a single gene with clearcut 1:1 orthologs in the other fully sequenced yeast genomes examined. Considering the sequence divergence of these RS and cognate tRNA species from their *S. cerevisiae* (*SACE*) counterparts over increasing phylogetic distances [8], we selected *Y. lipolytica* (*YALI*) to perform the experiments.

## Supplementary Note 2: Construction of transgenic *S. cerevisiae* strains bearing *Y. lipolytica* tRNA synthetases

Each of the six *S. cerevisiae* genes selected was replaced in such a way that the *Y. lipolytica* ortholog was under the control of the normal *S. cerevisiae* promoter (complete replacement of CDS from initiator to stop codons), as illustrated by Supplementary Fig. 1. This was done by integrative transformation in the diploid strain BYAT290 (Supplementary Table 1) of artificial DNA cassettes containing the KanMX gene for selection of transformant colonies on YPD medium containing 200 μg/ml of G418. After subcloning and verification of the proper integration of transforming cassettes (using genomic digest hybridizations), the six transformed diploid strains obtained (all showed normal growth despite being heterozygous *YALI*-RS / *SACE*-RS) were sporulated and submitted to tetrad analysis. Four viable ascospores were obtained in tetrads of the Tyr-RS diploid transformant, with no major difference in colony size between the $Kan^R$ and $Kan^S$ segregants (carrying respectively the *YALI* transgene and the normal *SACE* gene). We concluded that the *Y. lipolytica* Tyr-RS can successfully replace the *S. cerevisiae* Tyr-RS, preventing the type of experiments planned in this work. The other five diploid transformants (Glu-RS, Trp-RS, Asn-RS, Lys-RS and Asp-RS) yielded only two viable ascospores in tetrads, all of the $Kan^S$ phenotype, hence also preventing the type of experiments planned in this work.

We, therefore, decided to repeat the sporulations after transformation of each of the five heterozygous strains by a replicative plasmid bearing the cognate tRNA gene of *Y. lipolytica* (Supplementary Table 1). Several constructs were assayed (not shown) after which we repeatedly obtained viable $Kan^R$ haploid segregants in tetrads from the heterozygous diploids for the Asn-RS and Lys-RS gene replacements (respectively, BYAT521 transformed with plasmid pAT260 and BYAT526 transformed with plasmids pAT270 or pAT272). These segregants exhibited a very severe growth defect (Supplementary Fig. 2) but were able to propagate indefinitely, indicating that the *Y. lipolytica* Asn-RS and Lys-RS can, indeed, replace their *S. cerevisiae* orthologs for haploid cell survival, albeit with much reduced efficiency. The spontaneous plasmid loss observed in some slow growing $Kan^R$ haploid clones ($Ura^-$ phenotype) further demonstrated that the presence of the cognate *Y. lipolytica* tRNA gene was not necessary for survival after ascopore germination (note, however, the moderately larger colony size when plasmids were present). The unlikely hypothesis that the plasmid-borne *Y. lipolytica* tRNA genes might have integrated the chromosomes prior to

plasmid loss, hence explaining survival, was eliminated by analysis of genome sequence data (Methods).

After identification of mating-type and auxotrophic markers of the tetrad segregants, appropriate Kan$^{R}$ haploid strains were mated to produce homozygous diploids in which both *S. cerevisiae* alleles of either the Asn-RS or the Lys-RS gene were replaced by their *Y. lipolytica* orthologs (Supplementary Table 1). Like their haploid parents, these strains (BYAT580, BYAT581 and BYAT583) showed extremely reduced growth rates on YPD medium, but proved able of indefinite mitotic propagation.

## Supplementary Note 3: Functional aspects and phenotypic characterization of transgenic strains

The diploid strain BYAT580 bears two copies of the 586 codon-long *YALI0E05005g* gene of *Y. lipolytica* in place of the normal 555 codon-long *YHR019c* (*DED81*) gene of *S. cerevisiae*, corresponding to an orthologous *in situ* replacement of the Asn-RS gene on chromosome VIII. The two proteins share 66.7 % amino-acid identity (Supplementary Fig. 1). This strain originally contained the replicative plasmid pAT260 used for the initial construction (above), but plasmid-less derivatives were subsequently obtained. The diploid strains BYAT581 and BYAT583 bears two copies of the 583 codon-long *YALI0F16291g* gene of *Y. lipolytica* in place of the normal 592 codon-long *YDR037w* (*KRS1*) gene of *S. cerevisiae*, corresponding to an orthologous *in situ* replacement of the Lys-RS gene on chromosome IV. The two proteins share 67.5 % amino-acid identity (Supplementary Fig. 1).

The viability of these strains in absence of any *Y. lipolytica* tRNA gene demonstrated that the *YALI* Asn-RS and the *YALI* Lys-RS enzymes were able to charge tRNA molecules of *S. cerevisiae* with enough fidelity and efficacy for cell survival and accomplishment of mitotic cell cycles (as well as meiosis and sporulation). The genome of *S. cerevisiae* contains a total of 171,690 Asn codons (AAU + AAC) translated by a single isoacceptor (tRNA-Asn $^{[GUU]}$) encoded by 10 paralogous genes, and 206,734 Lys codons (AAA + AAG) translated by two isoacceptors (tRNA-Lys $^{[CUU]}$ and tRNA-Lys $^{[UUU]}$) encoded, respectively, by 14 and 7 paralogous genes. After intron elimination, the tRNA-Asn $^{[GUU]}$ molecules of *S. cerevisiae* differ from their *Y. lipolytica* counterparts by 11 substitutions and 1 indel (15% sequence divergence) [7]. Similarly, tRNA-Lys $^{[CUU]}$ and tRNA-Lys $^{[UUU]}$ molecules differ, respectively, by 17 substitutions and 1 indel and by 11 substitutions (24% and 15 % sequence divergences).

The severe growth rate reduction of BYAT580, BYAT581 and BYAT583 suggests that they experienced difficulties in protein synthesis and may be under severe stress as expected from a poor adaptation of the *Y. lipolytica* tRNA synthetases to the pools of cognate *S. cerevisae* tRNA molecules. To test this, we compared their transcriptomes with those of their normal-growing heterozygous parental strains that kept one copy of the *SACE* RS genes (Supplementary Fig. 3). A significant overexpression (4 - 42 times, average 16 times) was observed for all the seven genes of the lysine biosynthesis pathway (dispersed throughout the genome) in absence of any *SACE* Lys-RS allele. Overexpression also occurred for the transcriptional activator of this pathway. Less demonstrative results could be obtained in the absence of any *SACE* Asn-RS allele due to the smaller number of genes specifically involved in the asparagine biosynthesis pathway, but the two genes encoding the conversion of aspartate to asparagine were overexpressed 3 - 6 times. The smaller factor obtained here can be accounted for by the fact that strain BYAT580-0 already contained a low level

amplification of the *YALI* Asn-RS gene and was partially restored phenotypically (see text and Supplementary Fig. 10).

In conclusion, although we did not directly verify the amount of uncharged tRNA molecules in our transgenic strains or their degree of amino-acid misincorporation into proteins, their transcriptome patterns strongly suggest activation of the general amino-acid control pathway [9] symptomatic of an excess of uncharged tRNA molecules. The phenotypic restoration of BYAT580, BYAT581 and BYAT583 subclones to normal growth when these strains were transformed by plasmids bearing the normal *SACE* RS-genes (Supplementary Fig. 10) directly confirmed that their severe fitness reduction resulted solely from the orthologous replacements of the RS genes in absence of other accidental mutational change that might have occurred elsewhere in the genomes during strain construction. This conclusion is fully corroborated by complete genome sequencing (Supplementary Table 2). In particular, we did not observe any mutation in tRNA anticodons as reported in other experiments [10].

## Supplementary References


1. Dujon, B. *et al*. Genome evolution in yeasts. *Nature* **430**, 35-44 (2004).
2. Marck, C. DNA Strider: a “C” program for the analysis of DNA and protein sequences on the Apple Macintosch family of computers. *Nuc. Acids Res*. **16**, 1829-1836 (1988).
3. Christianson T.W. *et al.,* Multifunctional yeast high-copy number shuttle vectors. *Gene* **110**, 119-122 (1992).
4. Milne I. *et al.* Tablet next generation sequence assembly visualization. *Bioinformatics* **26**, 401-402 (2010).
5. Brachmann C.B., *et al.* Designer deletion strains derived from *Saccharomyces cerevisiae* S288C: a useful set of strains and plasmids for PCR-mediated gene disruption and other applications. *Yeast* **14**, 115-132 (1998).
6. Engel, SR. *et al.* The reference genome sequence of *Saccharomyces cerevisiae*: then and now. *G3* **4**:389-398 (2014).
7. Marck, C. *et al.* The RNA polymerase III-dependent family of genes in hemiascomycetes: comparative RNomics, decoding strategies, transcription and evolutionary implications. *Nuc. Acids Res*. **34**, 1816-1835 (2006).
8. Dujon, B. Yeast evolutionary genomics. *Nature Reviews Genetics* **11**, 512-524 (2010).
9. Hinnebusch, A.G. Translational regulation of *GCN4* and the general amino-acid control of yeast. *Ann. Rev. Microbiol.* **59**, 407-450 (2005).
10. Yona, A.H. *et al*. tRNA genes rapidly change in evolution to meet novel translational demands *eLIFE* **2**, e01339 (2013).
